# An Elite Haplotype of Nitrogen‐Use‐Efficiency Gene *LHT5* Enhances Salt Tolerance in Rice

**DOI:** 10.1111/pbi.70584

**Published:** 2026-02-06

**Authors:** Saisai Wang, Xingzhou Jiang, Gaoming Chen, Wei Wu, Chen Xu, Mingyu Du, Shuji Xiang, Xinran Cheng, Yunlu Tian, Junjie Tan, Chunming Wang, Jianmin Wan

**Affiliations:** ^1^ State Key Laboratory of Crop Genetics & Germplasm Enhancement and Utilization, Nanjing Agricultural University, Jiangsu Zhongshan Biological Breeding Laboratory Nanjing China; ^2^ Jiangsu Collaborative Innovation Center for Modern Crop Production, Southern Japonica Rice R&D Corporation Ltd Nanjing China; ^3^ State Key Laboratory of Crop Gene Resources and Breeding, Institute of Crop Sciences, Chinese Academy of Agricultural Sciences Beijing China

**Keywords:** lysine‐histidine‐type transporter, nitrogen use efficiency, *Oryza sativa* L., salt tolerance, superior allele

## Abstract

Amino acids serve as fundamental building blocks and signalling molecules in plants, orchestrating stress adaptation mechanisms against diverse biotic and abiotic environmental challenges. However, the mechanism by which plants alter their nutrient metabolism processes to coordinate nitrogen use efficiency (NUE) and salt tolerance remains elusive. Here, we identified a *Lysine‐Histidine‐type transporter 5* (*LHT5*) gene through genome‐wide association studies (GWAS) that enhances NUE via amino acid accumulation regulation. Further research showed that OsLHT5 also confers salt tolerance in rice by promoting proline biosynthesis through transcriptional upregulation of *OsP5CS1* and *OsP5CS2* genes, thereby increasing cellular proline levels for osmotic adjustment. Notably, we identified a functionally critical 30‐bp deletion in the *OsLHT5* coding region, designated as the elite haplotype *LHT5* ^*HapA*^, which substantially enhances amino acid transport capacity and consequently improves both NUE and salt tolerance. Functional validation demonstrated that overexpression of *LHT5* ^*HapA*^ significantly increases amino acid content, nitrogen accumulation, grain yield and salt stress tolerance compared to the wildtype allele. This study establishes a novel molecular framework linking amino acid transport to the coordination of nutrient utilisation and stress tolerance, offering valuable genetic resources and breeding strategies for developing climate‐resilient rice cultivars with enhanced productivity under both optimal and saline conditions.

## 1 Introduction

Salinity stress gradually diminishes plant growth and productivity, posing a significant threat to global agricultural sustainability. In response, plants have evolved intricate signalling pathways to combat salt toxicity, including ion homeostasis maintenance, osmotic adjustment and reactive oxygen species (ROS) detoxification (Yu et al. 2023). However, in saline field environments, nutrient availability is consistently limited due to poor soil conditions, consequently leading to reductions in plant growth and impaired nitrogen use efficiency (NUE) (Deng et al. 2022). Despite considerable advances in elucidating salt stress signalling pathways, the intricate interplay between plant nutrition and salt tolerance mechanisms remains a crucial yet often overlooked area.

Nitrogen serves as an essential macronutrient driving various physiological and biochemical processes in plants, playing a critical role in their growth and development. Upon absorption by plant roots, inorganic nitrogen undergoes assimilation into amino acids, which function as fundamental building blocks for protein synthesis and serve as the predominant form of long‐distance nitrogen transport from source to sink tissues (Tegeder and Masclaux‐Daubresse 2017). These amino acids are indispensable for the development of roots, leaves, flowers and seeds, while also contributing significantly to stress tolerance (Deng et al. 2022; Guo et al. 2020; Jiang et al. 2024; The et al. 2024; Xie et al. 2023). Our previous work demonstrated that OsGATA8 represses ammonium uptake by suppressing the expression of *OsAMT3.2*, an ammonium transporter in rice roots (Wu et al. 2024). After ammonium is taken up by OsAMT3.2 in rice, it must be efficiently assimilated into amino acids to support essential physiological processes, including tiller development.

*Amino acid transporter (AAT)* genes have been characterised across diverse plant species, including *Arabidopsis thaliana* (Tegeder 2012), rice (Zhao et al. 2012), soybean (Cheng et al. 2016), wheat (Wan et al. 2017) and potato (Ma et al. 2016). The *LHT (Lysine‐Histidine‐type Transporter)* gene family is expressed in multiple plant organs, including roots, leaves, and flowers, and plays dual roles in mediating amino acid uptake from soil and facilitating their subsequent transport and distribution within plants (Hirner et al. 2006). AtLHT1, the first characterised member of this family, functions in amino acid absorption and facilitates amino acid transport into mesophyll cells. Overexpression of *AtLHT1* enhances NUE under low‐nitrogen environments (Hirner et al. 2006; Svennerstam et al. 2007). In rice, mutation of *OsLHT1* leads to early senescence, rust‐red leaf spots, accumulation of ROS, accumulation, defence gene activation, and elevated jasmonic acid (JA) and salicylic acid (SA) biosynthesis. Interestingly, *OsLHT1* dysfunction also confers resistance to rice blast fungus infection (Guo et al. 2023). However, the mechanism underlying salinity tolerance by the *LHT* gene family remains unexplored.

In this study, we conducted GWAS on nitrogen‐efficient traits and identified *OsLHT5* as a candidate gene significantly associated with effective panicle number ratio (EPNR). Our findings demonstrate that *OsLHT5* positively regulates both amino acid content and effective panicle number, consequently influencing grain yield and NUE. Moreover, overexpression of the coding sequence (CDS) of the elite haplotype *LHT5* ^*HapA*^ significantly increased amino acid levels, including proline, and simultaneously enhanced both NUE and salt tolerance in rice. Thus, our findings reveal a previously unrecognised dual function of elite allele *OsLHT5* in coordinating plant growth and salt tolerance, positioning this gene as a promising target for developing crop varieties with improved NUE and enhanced salt tolerance.

## 2 Results

### 2.1 Identification and Functional Analysis of *OsLHT5*

To identify novel factors regulating NUE in rice, we first measured NUE‐related agronomic traits in 175 core varieties from the 3 K Rice Genomes Project (Wang et al. 2018) which were grown in low‐nitrogen (LN) and high‐nitrogen (HN) fields. These traits included the plant height ratio (PHR, calculated as PH‐LN/PH‐HN) and effective panicle number ratio (EPNR, calculated as EPN‐LN/EPN‐HN) (Figure S1) (Tang et al. 2019).

**FIGURE 1:**
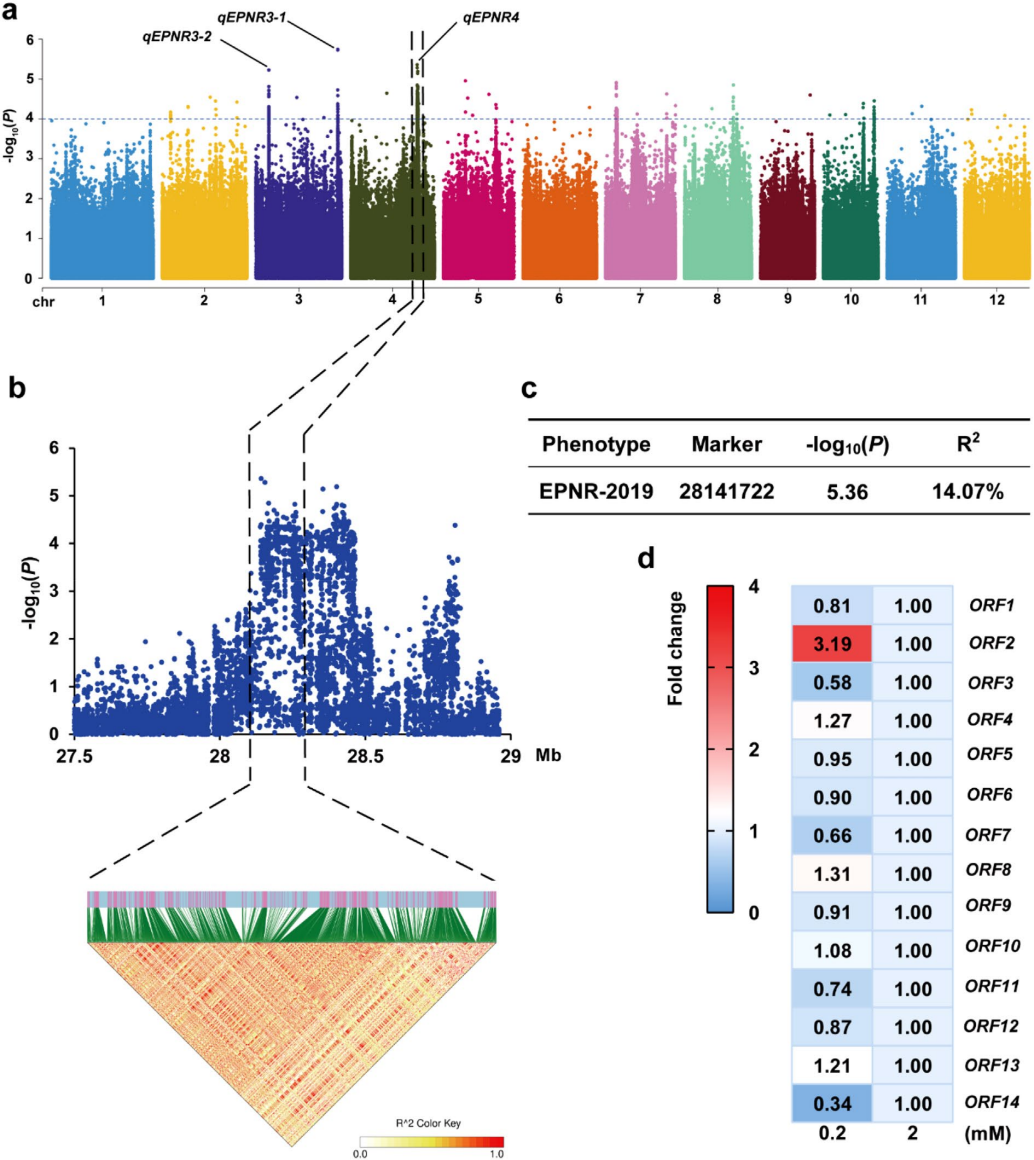
GWAS and identification of *OsLHT5* on chromosome 4. (a) Manhattan plot for EPNR and QTL loci. (b) Local Manhattan plot (top) and LD heatmap (bottom) of *qEPNR4*. Black dashed lines indicate the candidate region for the peak. (c) Leader SNP (chr4:28141722), LOD value, and PVE (%) of *qEPNR4*. (d) Heat map of the relative expression levels of 14 *ORFs* within the LD block under low nitrogen (LN) induction. The relative expression level of each gene under 2 mM NH_4_NO_3_ treatment was set as 1.00.

We performed a GWAS using 3 073 537 single nucleotide polymorphisms (SNPs), which showed non‐random distribution across all chromosomes (Figure S2a). Population structure analysis based on these SNPs revealed that the cross‐validation (CV) error was lowest when the model parameter *K* = 7, thus dividing the 175 accessions into seven subgroups (Figure S2b,c). The statistical analysis revealed that the *p*‐values for three QTLs (*qEPNR3‐1*, *qEPNR3‐2* and *qEPNR4*) were below the significance threshold of 10^−5^ (Figure 1a, Figure S3a). The QTL *qEPNR3‐1* is characterised by a relatively small linkage interval (3 kb). The SNPs within this region are intergenic and do not overlap with any open reading frame (ORF). For *qEPNR4* with the second smallest *p*‐value, a major QTL within a linkage disequilibrium (LD) block was mapped to chromosome 4 (Figure 1b,c). To identify potential candidate genes for this QTL (Figure S3b), we analysed the expression patterns of these 14 *ORFs* in the cultivar Nipponbare (Figure S3b). Among these candidates, *ORF2* exhibited the most significant upregulation in response to LN induction. Further annotation analysis revealed that *ORF2*, which encodes a Lysine‐Histidine‐type Transporter 5 (LHT5), was the candidate gene contributing to NUE within this LD block (Figure 1d). OsLHT5 shares high homology with OsLHT1 and contains the same conserved domains characteristic of the LHT family, suggesting similar amino acid transport capabilities (Zhao et al. 2012).

Furthermore, quantitative PCR (qPCR) analysis was conducted to verify the tissue‐specific expression profile of *OsLHT5* in rice. We found that *OsLHT5* was highly expressed in roots, leaf blades and post‐flowering panicles (Figure S3c). To determine the subcellular localisation of OsLHT5, we fused green fluorescent protein (GFP) to the C‐terminus of OsLHT5 and transiently expressed this fusion protein in rice protoplasts. Confocal microscopy analysis confirmed that OsLHT5 was predominantly localised to the plasma membrane (Figure S3d).

### 2.2 *OsLHT5* Positively Regulates NUE by Improving Amino Acid Accumulation

**FIGURE 2:**
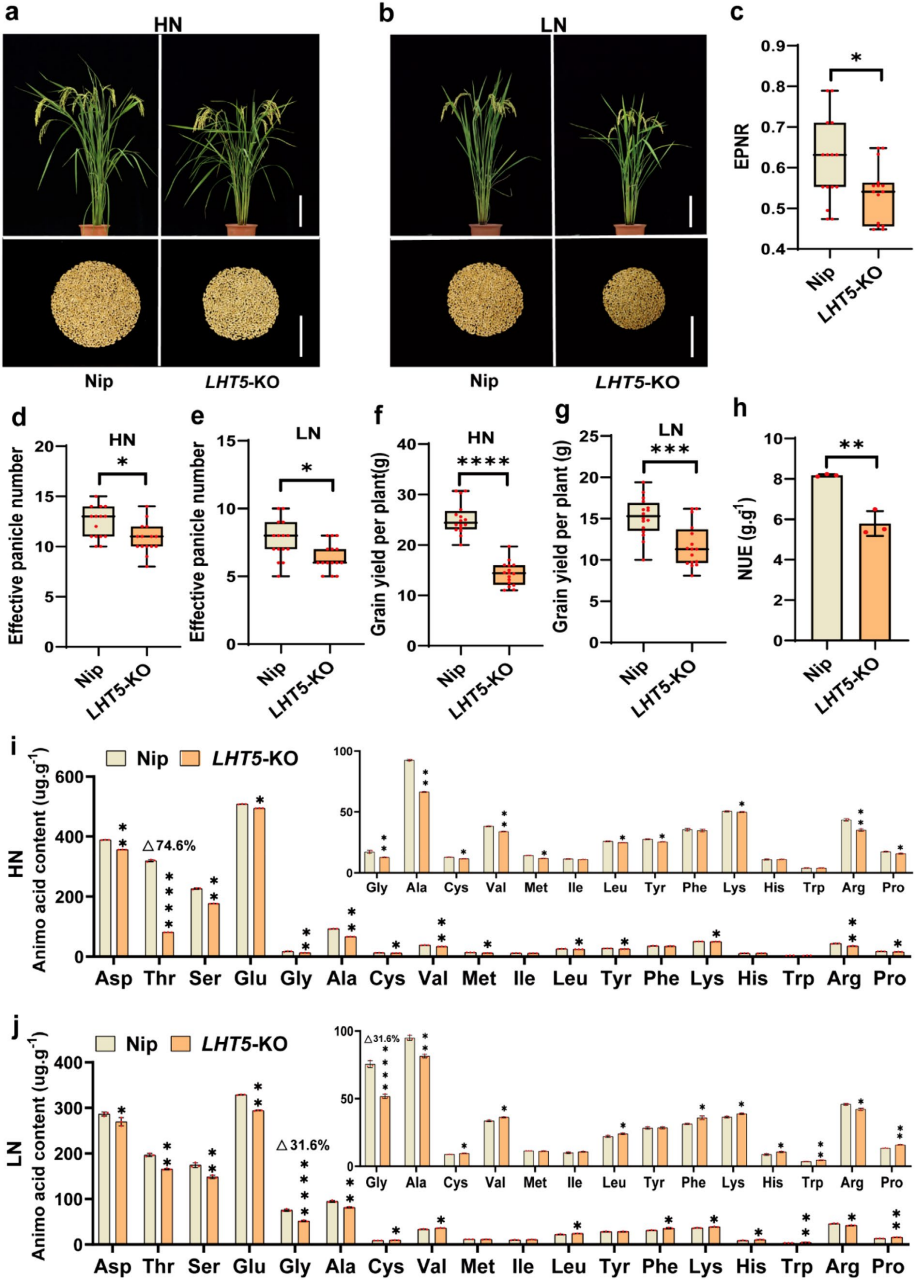
*OsLHT5* positively regulates amino acid content and NUE in rice. (a, b) Phenotypes of wild‐type cultivar Nipponbare and *LHT5‐*KO (Knockout) mutant grown in high‐nitrogen and low‐nitrogen fields at 90‐d old. Scale bar = 18 cm. Grains were taken after harvest. Scale bar = 10 cm. (c–g) Phenotypic analysis of effective panicle number ratio (c), effective panicle number (d, e) and grain yield per plant (f, g), of wild‐type cultivar Nipponbare and *LHT5‐*KO mutant plants under high‐ and low‐nitrogen conditions. (h) NUE (grain yield in HN–grain yield in LN plot (6 × 8) / N supply) of WT and *LHT5‐*KO mutant, *n* = 3. Bars represent the mean ± SD. (i, j) Single concentration of amino acids in shoot of WT and *LHT5‐*KO mutant seedlings treated with 0.2 and 2 mM NH_4_NO_3_ for 7 days, *n* = 3. Bars represent the mean ± SD. Statistically significant differences are indicated by different letters. *, **, and ***, **** of *t*‐test indicate significant differences at *p* < 0.05, *p* < 0.01, *p* < 0.001, *p* < 0.0001.

To validate the functional role of *OsLHT5*, a CRISPR/Cas9 knockout line (*LHT5*‐KO) was generated in Nipponbare (Nip) background (Figure S4a). The effective panicle number (EPN), effective panicle number ratio (EPNR), and grain yield per plant (YPP) of the *LHT5*‐KO line were measured under low‐nitrogen (LN) and high‐nitrogen (HN) field conditions (Figure 2a,b). Compared with the wild‐type Nip, the *LHT5*‐KO line exhibited significant reductions in EPNR (Figure 2c), EPN (Figure 2d,e), YPP (Figure 2f,g) and NUE (Figure 2h). To further evaluate nitrogen utilisation efficiency, wild‐type seedlings and the *LHT5*‐KO lines were treated with high (2 mM) and low (0.2 mM) NH_4_NO_3_ for 14 days (Figure S4b). Additionally, phenotypic analysis at the seedling stage revealed that *LHT5*‐KO plants had lower fresh weight (FW), dry weight (DW), and nitrogen accumulation than Nip under both LN and HN conditions (Figure S4c–h). Collectively, these results indicate that *OsLHT5* plays a critical role in regulating NUE.

To investigate the mechanisms of *OsLHT5* on NUE in rice, we quantified the concentrations of individual amino acids (Figure 2i,j) and total amino acids (Figure S4i,j) in seedling shoots of Nip and *LHT5*‐KO lines grown under LN and HN conditions. The results demonstrated that both individual and total amino acid concentrations were significantly decreased in *LHT5*‐KO plants compared to the wild type (WT). Notably, threonine exhibited the greatest reduction of 74.6% under the HN condition, whereas glycine showed the most pronounced decrease of 31.6% under the LN condition. These findings suggest that OsLHT5 influences the content of multiple amino acids, especially the neutral amino acids.

### 2.3 Variation in *OsLHT5* Affects Amino Acid Levels and Nitrogen Utilisation

**FIGURE 3:**
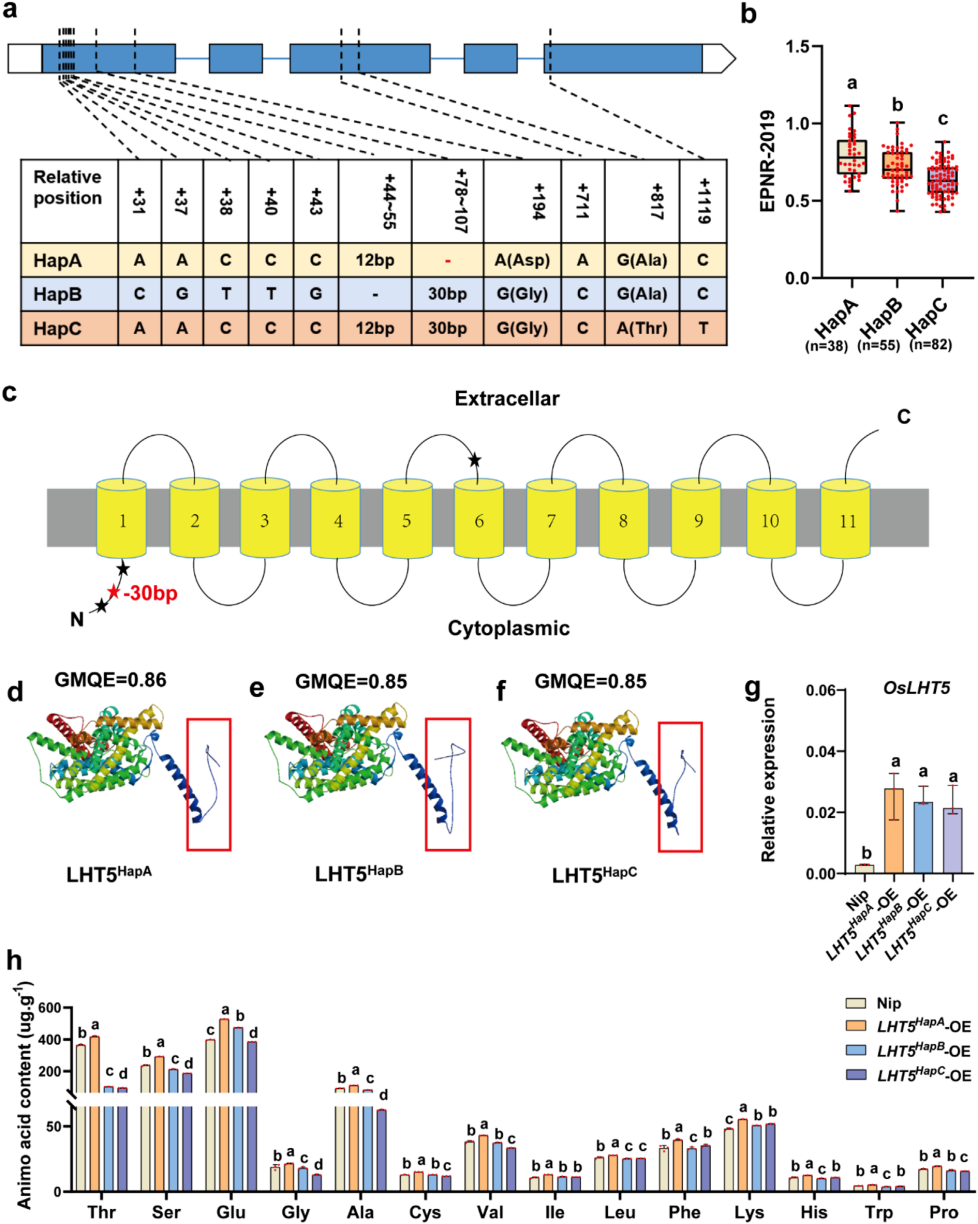
The elite haplotype of *OsLHT5* is associated with elevated EPNR and amino acid content. (a) Sequence analysis of the *OsLHT5* coding sequence in 175 rice accession varieties. The white box represents untranslated regions (UTRs), blue lines represent introns, and the light blue box represents coding regions. (b) Comparison of EPNR in three haplotypes of *OsLHT5*. (c) The transmembrane domain of OsLHT5 was predicted using the TMHMM website (http://www.cbs.dtu.dk/). Asterisks indicate indel and missense SNP sites within OsLHT5 protein. (d–f) The three‐dimensional structures of OsLHT5 proteins with different haplotypes were predicted using SWISS‐MODEL (https://swissmodel.expasy.org/). (g) The expression levels (*n* = 3) of WT and *OsLHT5* overexpression lines containing different haplotype CDS in the seeding stage. Bars represent the mean ± SD. (h) Amino acid concentration (*n* = 3) of WT and OsLHT5 overexpression lines containing different haplotype CDS in seeding stage. Bars represent the mean ± SD. Statistical significance was calculated by one‐way ANOVA with Duncan's multiple range test (*p* < 0.05). Statistically significant differences are indicated by different letters.

Sequence analysis identified nine SNPs and two indels within the coding sequences (CDS) of *OsLHT5*. Based on missense SNPs and indels in the coding region, *OsLHT5* was classified into three haplotypes (Figure 3a). Phenotypic analysis revealed that *LHT5* ^*HapA*^ represents the elite haplotype, exhibiting a significantly higher EPNR and EPN compared to *LHT5* ^*HapB*^ and *LHT5* ^*HapC*^ haplotypes (Figure 3b, Figure S5a,b).

To explore the functional implications of these two missense SNPs (+194 and +817) and two indels (+44–55 and +78–107) in the coding region, we predicted the transmembrane domains and three‐dimensional structure of OsLHT5 using TMHMM (http://www.cbs.dtu.dk/) and SWISS‐MODEL (https://swissmodel.expasy.org/). Our analysis revealed that the 12‐bp indel and 30‐bp indel, as well as the +194G → A substitution located in the N‐terminus of LHT5 protein (Figure 3c), altered the three‐dimensional structure of OsLHT5 (Figure 3d–f). To validate the functional significance of these structural variations, we generated overexpression (OE) lines of the three haplotypes in the Nipponbare background (*LHT5* ^*HapA*^‐OE, *LHT5* ^*HapB*^‐OE, and *LHT5* ^*HapC*^‐OE) and identified OE lines exhibiting comparable expression levels of *OsLHT5*, which were significantly higher than those of the wild type (Figure 3g). Amino acid profiling of seedling shoots demonstrated that *LHT5* ^*HapA*^‐OE lines accumulated significantly higher concentrations of individual amino acids compared to WT, *LHT5* ^*HapB*^‐OE and *LHT5* ^*HapC*^‐OE lines (Figure 3h), confirming the superior transport capacity of the elite haplotype.

**FIGURE 4:**
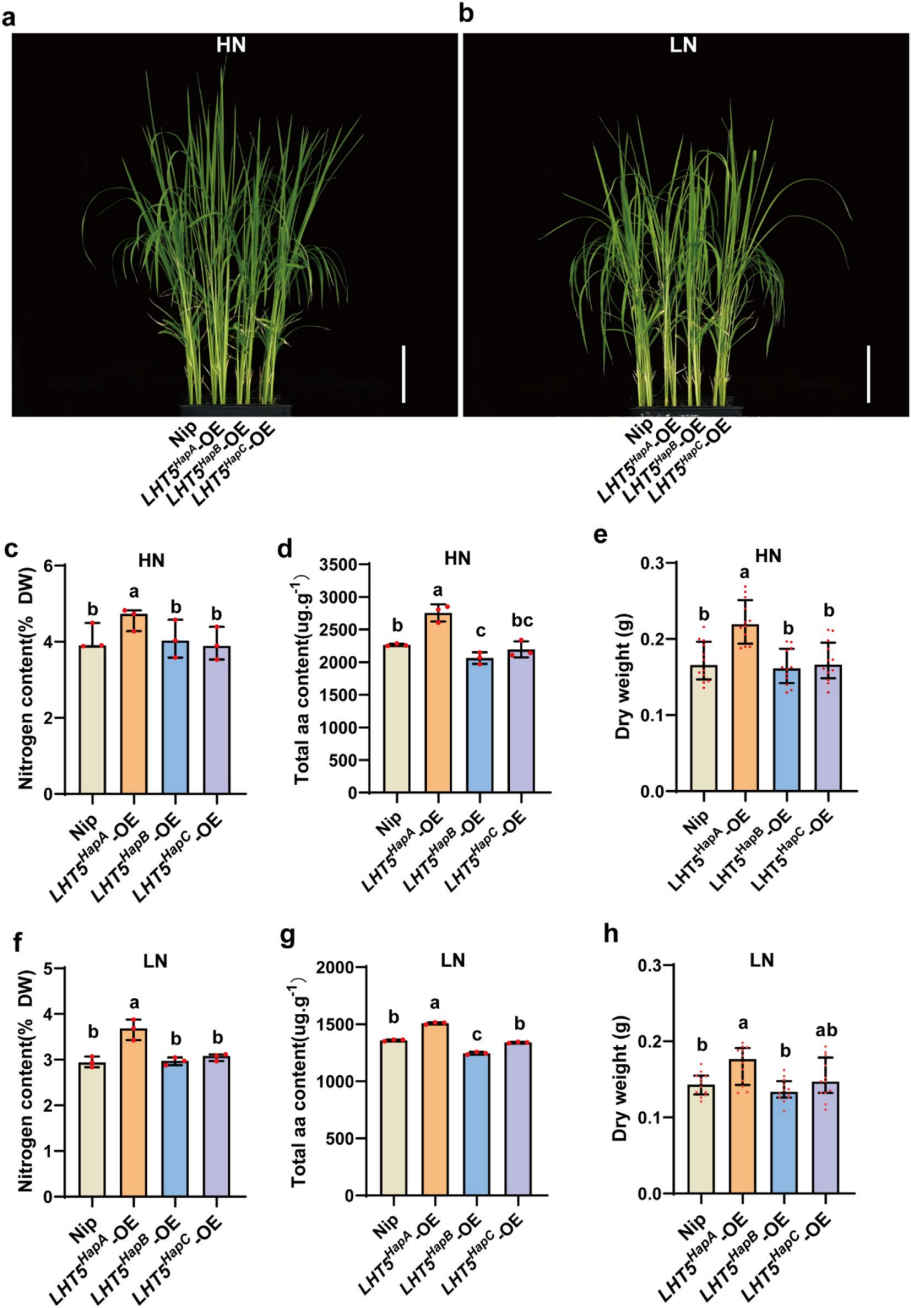
The elite haplotypes of *OsLHT5* ^*HapA*^ enhance nitrogen and amino acid content. (a, b) Phenotypes of Nipponbare and *OsLHT5* overexpression lines containing different haplotype CDS grown in high‐nitrogen and low‐nitrogen (2 mM and 0.2 mM NH_4_NO_3_) conditions at 14‐d old. Scale bar = 5 cm. (c–h) Phenotypic analysis of nitrogen content (*n* = 3), total free amino acid concentration (*n* = 3), dry weight (*n* ≥ 9) of Nipponbare and *OsLHT5* overexpression lines containing different haplotype CDS under high‐ and low‐nitrogen conditions. Bars represent the mean ± SD. Statistical significance was calculated by one‐way ANOVA with Duncan's multiple range test (*p* < 0.05). Statistically significant differences are indicated by different letters.

To further evaluate nitrogen utilisation efficiency, wild‐type seedlings and the three *LHT5*‐OE lines were treated with high (2 mM) and low (0.2 mM) NH_4_NO_3_ for 14 days (Figure 4a,b). Under both NH_4_NO_3_ treatments, *LHT5* ^*HapA*^‐OE plants consistently exhibited significantly higher dry weight (DW), total nitrogen content and amino acid content compared with the wild type, *LHT5* ^*HapB*^‐OE, and *LHT5* ^*HapC*^‐OE plants (Figure 4c–h). Collectively, these results indicate that variations in *LHT5* ^*HapA*^ contribute to enhanced amino acid levels and nitrogen utilisation in rice.

**FIGURE 5:**
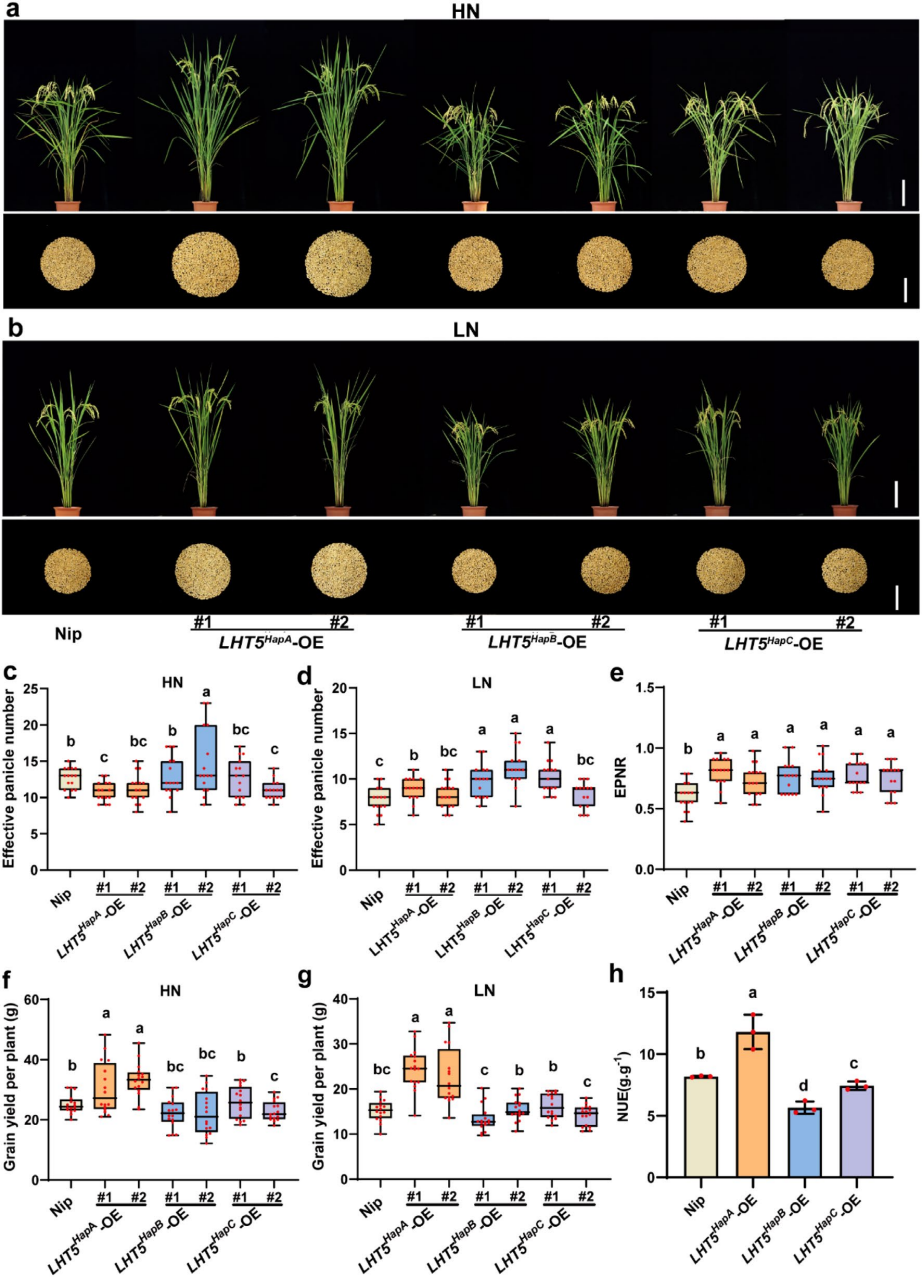
*OsLHT5* improves grain yield in high and low nitrogen conditions. (a, b) Phenotypes of Nipponbare and *OsLHT5* overexpression lines containing different haplotype CDS under LN and HN paddy fields at mature stages. Scale bar = 18 cm. Grains were taken after harvest. Scale bar = 10 cm. (c, d) Effective‐panicle number (*n* = 15) of Nipponbare and *OsLHT5* overexpression lines containing different haplotype CDS under HN (c) and LN conditions (d). Bars represent the mean ± SD. (e) Effective‐panicle number ratio (*n* = 15) of Nipponbare and *OsLHT5* overexpression lines containing different haplotype CDS. Bars represent the mean ± SD. (f, g) Grain yield per plant (*n* = 15) of Nipponbare and *OsLHT5* overexpression lines containing different haplotype CDS under HN (f) and LN conditions (g). Bars represent the mean ± SD. (h) NUE (grain yield in HN–grain yield in LN plot (6 × 8) / N supply) of Nipponbare and *OsLHT5* overexpression lines containing different haplotype CDS, *n* = 3. Bars represent the mean ± SD. Statistical significance was calculated by one‐way ANOVA with Duncan's multiple range test (*p* < 0.05). Statistically significant differences are indicated by different letters.

To further assess whether *LHT5* ^*HapA*^ has breeding potential for improving NUE in rice, we determined YPP, EPNR, yield per plot and NUE of wild‐type, *LHT5* ^*HapA*^‐OE, *LHT5* ^*HapB*^‐OE and *LHT5* ^*HapC*^‐OE lines under LN and HN fields (Figure 5a,b). At the mature stage, *LHT5* ^*HapA*^ lines not only showed higher EPN (Figure 5c,d), EPNR (Figure 5e) and YPP (Figure 5f,g) but also increased yield per plot (Figure S6a–c) and NUE (Figure 5h). These findings demonstrate that *LHT5* ^*HapA*^ confers superior agronomic traits and holds value for breeding applications.

### 2.4 Superior Allele *LHT5* ^*HapA*^ Enhances Salt Tolerance

**FIGURE 6:**
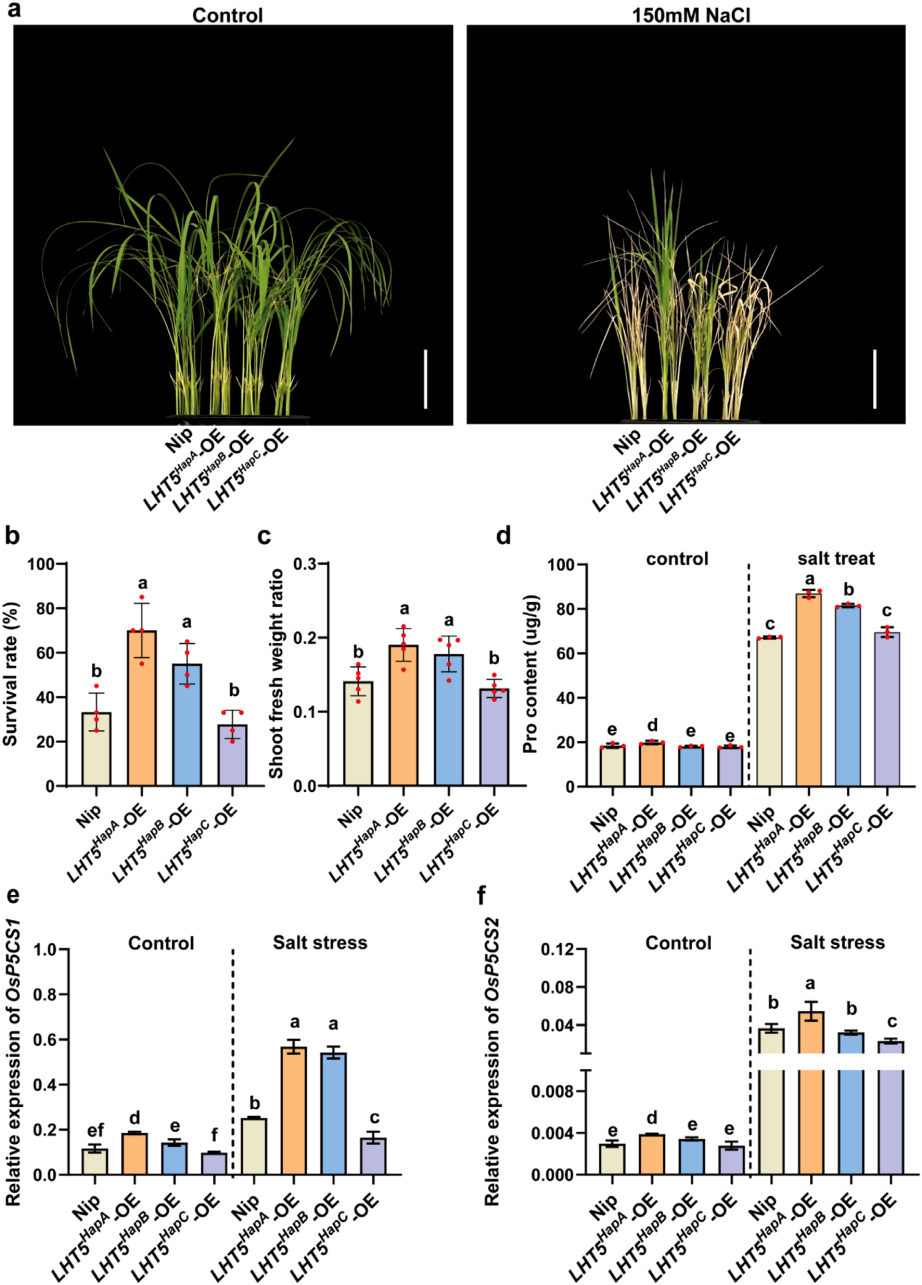
*OsLHT5* improves salt tolerance by elevating proline levels. (a) Phenotypes of Nipponbare and *OsLHT5* overexpression lines containing different haplotype CDS under 0 mM and 150 mM NaCl treatment for 10 days, Scale bar = 5 cm. (b) Survival rate of Nipponbare and *OsLHT5* overexpression lines containing different haplotype CDS under 150 mM NaCl treatment for 10 days (*n* = 4). (c) Shoot fresh weight ratio of the three plants of Nipponbare and *OsLHT5* overexpression lines containing different haplotype CDS under 150 mM NaCl treatment for 10 days (*n* = 5). (d) Proline content of Nipponbare and *OsLHT5* overexpression lines containing different haplotype CDS under 0 mM and 150 mM NaCl conditions (*n* = 3). (e and f) The relative expression of *OsP5CS1* (e) and *OsP5CS2* (f) in Nipponbare and *OsLHT5* overexpression lines containing different haplotype CDS under 0 mM and 150 mM NaCl treatment (*n* = 3). Statistical significance was calculated by one‐way ANOVA with Duncan's multiple range test (*p* < 0.05). Statistically significant differences are indicated by different letters.

Previous studies have demonstrated that proline accumulation serves as a critical mechanism for enhancing salt tolerance in rice (Nie et al. 2024). Given that our results demonstrated *OsLHT5* influences the content of multiple amino acids, including proline, we hypothesised that *OsLHT5* might improve salt tolerance through proline‐mediated osmoregulation. To test this hypothesis, we treated wild‐type, *LHT5* ^*HapA*^‐OE, *LHT5* ^*HapB*^‐OE and *LHT5* ^*HapC*^‐OE seedlings with 150 mM NaCl (Figure 6a). After 10 days of salt treatment, *LHT5* ^*HapA*^‐OE plants exhibited a significantly higher survival rate (SR) and shoot fresh weight ratio (SFWR) compared to WT and the other haplotype lines (Figure 6b,c). Consistent with this enhanced stress tolerance, amino acid analysis revealed that proline content in *LHT5* ^*HapA*^‐OE plants was significantly higher than in wild‐type, *LHT5* ^*HapB*^‐OE, and *LHT5* ^*HapC*^‐OE seedlings (Figure 6d). To elucidate the molecular mechanism underlying this proline accumulation, we examined the expression of *OsP5CS1* and *OsP5CS2*, two key genes encoding Δ1‐pyrroline‐5‐carboxylate synthase enzymes that catalyse the rate‐limiting step in proline biosynthesis. Quantitative expression analysis under normal and salt treatment conditions revealed that *LHT5* ^*HapA*^‐OE plants exhibited the highest relative expression levels of *OsP5CS1* and *OsP5CS2* compared to all other lines (Figure 6e,f). Collectively, these findings indicate that the elite haplotype *LHT5* ^*HapA*^ enhances salt tolerance through increasing the accumulation of proline.

### 2.5 A 30‐Bp Deletion in *LHT5* ^*HapA*^ Elite Haplotype Confers Higher NUE and Salt Tolerance

To identify the causal SNP underlying the superior phenotype of the *LHT5* ^*HapA*^ haplotype, we synthesised gene sequences harbouring either the 30‐bp deletion (positions 78–107) or the single‐base substitution (G → A position 194) within the *OsLHT5* coding region. Using these sequences, we generated overexpression lines in the Nipponbare background, designated as *LHT5* ^*30bp*^‐OE and *LHT5* ^*G‐A*^‐OE, respectively.

**FIGURE 7:**
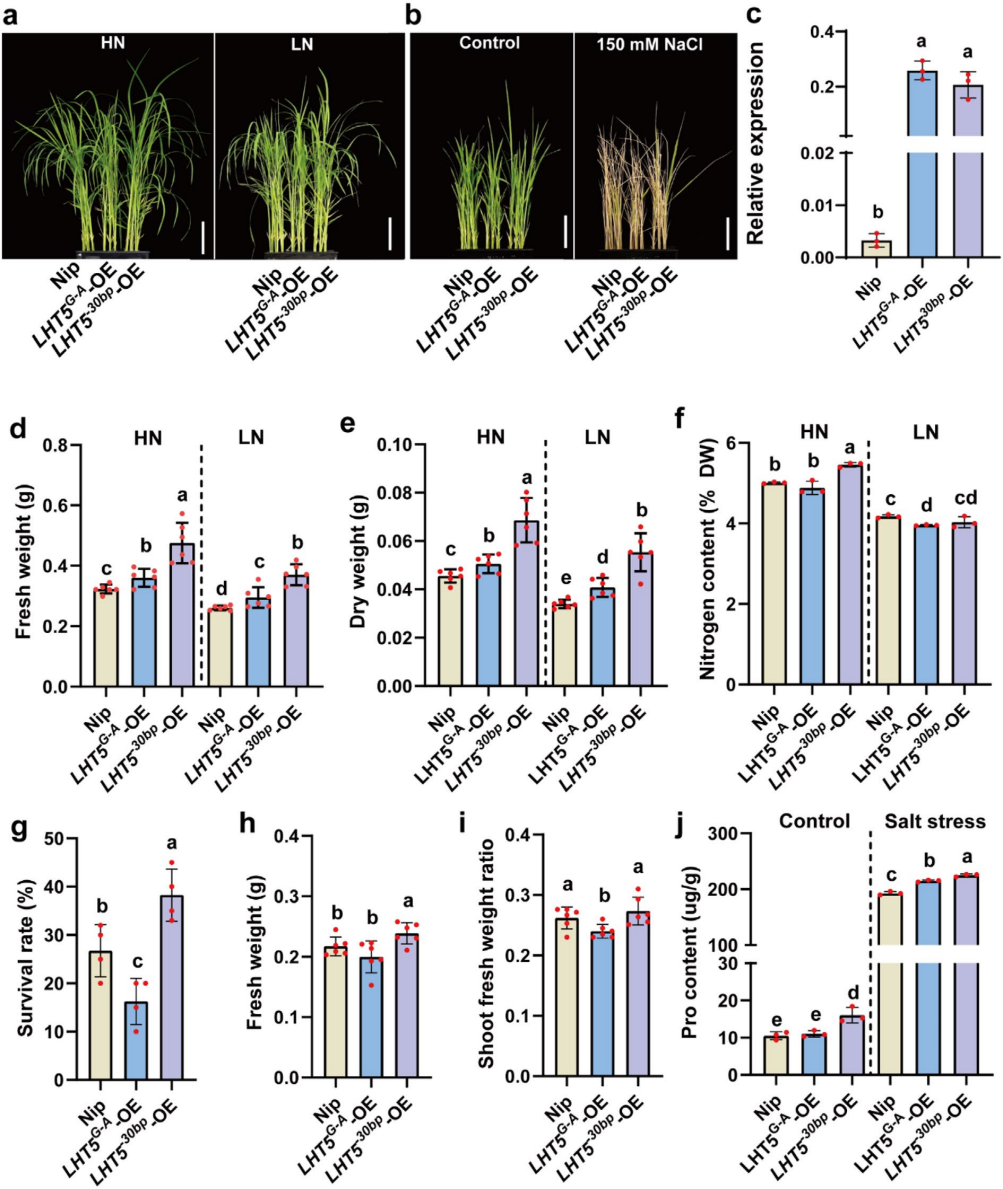
Phenotypes of 30‐bp deletion and G‐A substitution in *LHT5* ^*HapA*^. (a) Phenotypes of *LHT5* ^*G‐A*^‐OE and *LHT5* ^*30bp*^‐OE lines were plants grown on HN and LN conditions (2 mM and 0.2 mM NH_4_NO_3_) 14 days, Scale bar = 5 cm. (b) Phenotypes of *LHT5* ^*G‐A*^‐OE and *LHT5* ^*30bp*^‐OE lines plants grown on nutrient solution, supplemented with 150 mM NaCl for 10 days, Scale bar = 5 cm. (c) The expression levels (*n* = 3) of WT and *LHT5* ^*G‐A*^‐OE and *LHT5* ^*30bp*^‐OE lines in seeding stage. Bars represent the mean ± SD. (d–f) Phenotypic analysis of nitrogen content (*n* = 3), fresh weight (*n* = 6), dry weight (*n* = 6) of Nipponbare and *LHT5* ^*G‐A*^‐OE and *LHT5* ^*30bp*^‐OE lines under high‐ and low‐nitrogen conditions. (g–i) Survival rate (f), fresh weight (g) of the three plants and shoot fresh weight ratio of the three plants (h) of Nipponbare and *LHT5* ^*G‐A*^‐OE and *LHT5* ^*30bp*^‐OE lines under 150 mM NaCl treatment for 10 days (*n* = 6). (j) Proline content of Nipponbare and *LHT5* ^*G‐A*^‐OE and *LHT5* ^*30bp*^‐OE lines under 0 mM and 150 mM NaCl conditions (*n* = 3). Bars represent the mean ± SD. Statistical significance was calculated by one‐way ANOVA with Duncan's multiple range test (*p* < 0.05). Statistically significant differences are indicated by different letters.

To compare the effects of these two variations on NUE, we subjected wild‐type, *LHT5* ^*30bp*^‐OE and *LHT5* ^*G‐A*^‐OE seedlings to high (2 mM) and low (0.2 mM) NH_4_NO_3_ for 14 days (Figure 7a). We identified *LHT5* ^*30bp*^‐OE and *LHT5* ^*G‐A*^‐OE lines exhibiting comparable expression levels of *OsLHT5* (Figure 7c). Under both NH_4_NO_3_ treatments, *LHT5* ^*30bp*^‐OE plants consistently exhibited significantly higher dry weight, fresh weight and total nitrogen content compared to wild‐type and *LHT5* ^*G‐A*^‐OE plants (Figure 7d–f). These results indicate that the 30‐bp deletion variant confers higher nitrogen utilisation efficiency than the G → A substitution variant. To evaluate salt tolerance, we treated wild‐type, and *LHT5* ^***30bp***^‐OE and *LHT5* ^***G‐A***^‐OE seedlings with 150 mM NaCl (Figure 7b). After 150 mM NaCl treatment for 10 days, the *LHT5* ^***30bp***^‐OE plants exhibited higher SR, FW, SFWR and proline content compared to *LHT5* ^***G‐A***^‐OE and wild‐type plants (Figure 7g–j). These results suggest that *LHT5* ^***30bp***^ conferred higher salt tolerance than *LHT5* ^***G‐A***^. Collectively, these results demonstrate that the 30‐bp deletion in *LHT5* ^*HapA*^ coding sequence serves as the primary causal variant responsible for both improved NUE and enhanced salt tolerance.

## 3 Discussion

Plants absorb nitrogen and assimilate it into amino acids to support growth and adaptation to diverse biotic and abiotic stresses (Guo et al. 2023). In saline environments, nutrient availability is relatively limited, and exposure to salt stress leads to reduced plant growth and impaired NUE (Deng et al. 2022). This study identifies the elite haplotype *LHT5* ^*HapA*^, which simultaneously enhances both NUE and salt tolerance in rice. Specifically, *LHT5* ^*HapA*^ promotes nitrogen utilisation through enhanced amino acid accumulation, with a particular preference for the neutral amino acid glycine and threonine. The elevated amino acid level promotes increases in tiller number in rice, enhances the thousand‐grain weight of rice seeds, and consequently boosts rice yield while improving nitrogen use efficiency (NUE) in rice. Meanwhile, *LHT5* ^*HapA*^ upregulates the expression of key genes involved in proline synthesis, leading to increased proline accumulation, which is more pronounced under salt stress.

Plants have evolved complex signalling pathways to combat salt stress through multiple mechanisms, including ion homeostasis maintenance, osmotic adjustment and ROS scavenging (Yu et al. 2023). Based on this foundation, previous studies have elucidated several key molecular players in rice salt tolerance. For instance, our earlier work demonstrated that the microtubule protein OsTUB1 protects rice from salt stress toxicity by stabilising Na^+^ transport protein OsHKT1;5 under salt stress (Chen et al. 2022). Similarly, the transcription factor OsWRKY53 confers salt protection by regulating the expression of *OsMKK10.2/OsHKT1*;5 (Yu et al. 2023). Additionally, the late embryogenesis abundant protein LEA12 contributes to osmotic regulation and yield maintenance under salt stress (Ge et al. 2024). However, these established mechanisms primarily address direct salt toxicity while overlooking a critical aspect of plant performance in saline environments: the intersection of nutrition and stress tolerance. In saline field environments, nutrient availability is consistently limited due to deteriorated soil properties, creating a dual challenge where plants must simultaneously cope with salt toxicity and nutritional deficiency (Deng et al. 2022). Despite advances in understanding salt stress signalling pathways, the role of nutrient metabolism in salt adaptation has remained largely underexplored by plant biologists and agronomists. Our study addresses this by demonstrating how amino acid transport bridges nutritional efficiency and stress tolerance. Specifically, we reveal that OsLHT5 enhances rice salt tolerance through a dual mechanism: elevating amino acid levels to improve nutrient availability while simultaneously increasing proline accumulation for osmotic protection. This discovery demonstrates that nutritional and stress responses are coordinately regulated rather than functioning as independent pathways. These findings expand our understanding of salt tolerance in rice by revealing how plants integrate metabolic efficiency with stress adaptation through amino acid‐mediated mechanisms.

The enhanced transport of amino acids, coupled with the upregulated expression of proline biosynthesis‐related genes, collectively contributed to the elevated proline levels. On one hand, overexpression of *OsLHT5* ^*HapA*^ facilitated amino acid uptake, thereby supplying more precursors for proline synthesis. On the other hand, the activation of signalling pathways led to increased expression of *OsP5CS1* and *OsP5CS2*, which enhanced the enzymatic capacity for proline production. The synergistic interaction between these two mechanisms ultimately resulted in a marked accumulation of proline.

Regarding amino acid transport activity, the coding regions of OsLHT5^HapB^ and OsLHT5^HapC^ contain a 30‐bp sequence, and these two haplotypes exhibit relatively weak amino acid transport capacity, only transporting a small number of specific amino acids. In contrast, the coding region of OsLHT5^HapA^ lacks this 30‐bp sequence and shows significantly stronger capacity to transport a variety of amino acids (Figure 3h). Based on these observations, we hypothesise that the N‐terminal region of OsLHT5 may exert an inhibitory effect on its transport activity; when this region is deleted or truncated, the transport capacity of OsLHT5 is enhanced. OsLHT5^HapB^ and OsLHT5^HapC^ can increase the number of effective tillers in rice (Figure 5c,d), suggesting that these two haplotypes exert their functional effects during the reproductive growth stage of the crop.

**FIGURE 8:**
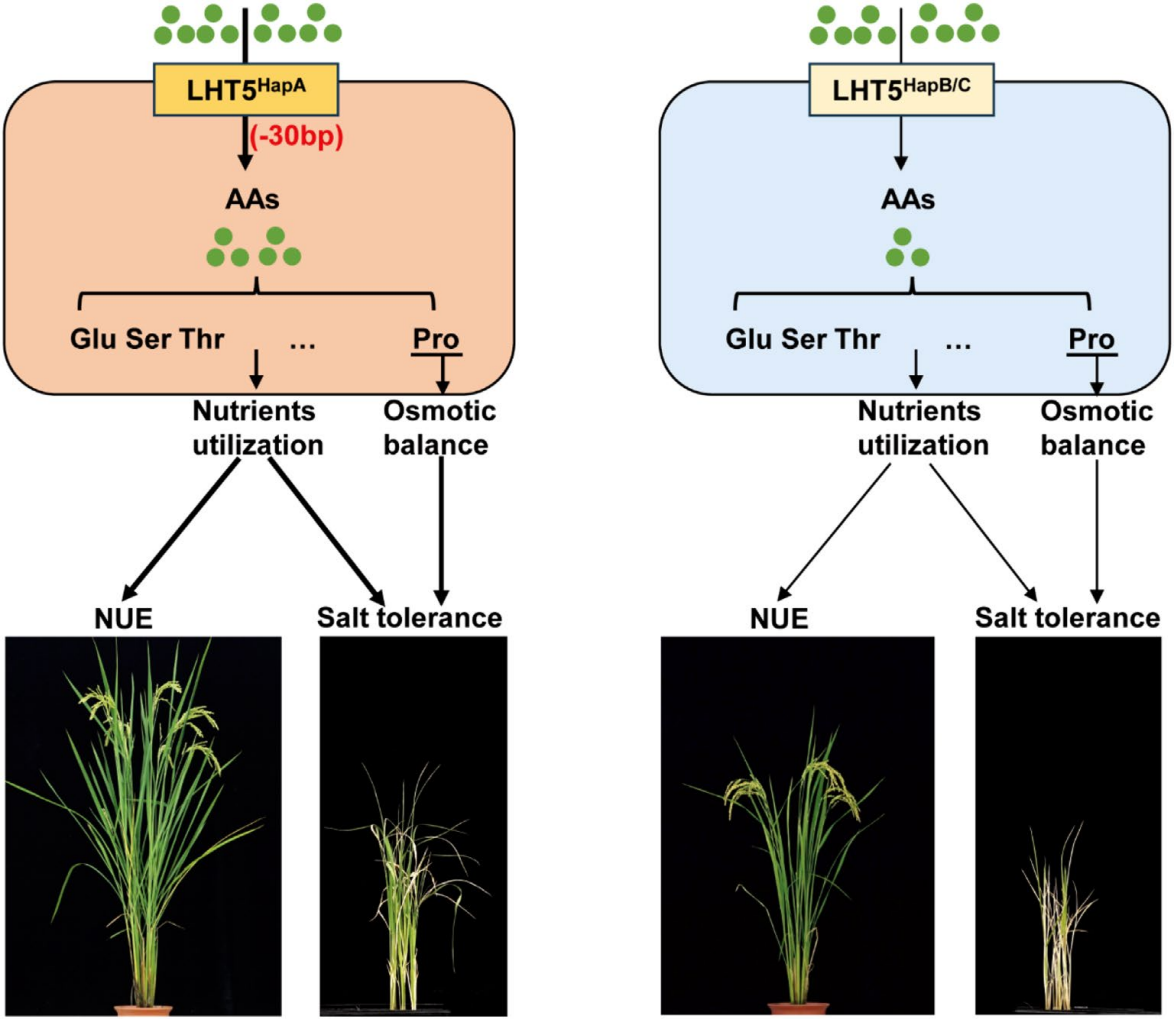
A proposed working model illustrating the role of *OsLHT5* in regulating rice NUE and salt tolerance. *OsLHT5* enhances nitrogen utilisation and salt tolerance by promoting the accumulation of multiple amino acids, while also improving salt tolerance through the regulation of proline levels. The 30‐bp deletion within the coding region of *LHT5* ^*HapA*^ leads to greater amino acid accumulation compared to *LHT5* ^*HapB*^ and *LHT5* ^*HapC*^ in rice. Collectively, the elite haplotype of the NUE‐related gene *OsLHT5* contributes to enhanced salt tolerance in rice.

Additionally, the advantageous haplotype *LHT5* ^*HapA*^ highlights it as an elite allele with substantial breeding value. Notably, the 30‐bp deletion within the coding region of *LHT5* ^*HapA*^ is responsible for the enhanced amino acid accumulation in rice (Figure 8). Moving forward, precise 30‐bp deletions mediated by targeted gene‐editing technologies could be utilised to generate valuable breeding materials. Experimental evidence indicates that the 30‐bp deletion is the causal SNP that makes *LHT5* ^*HapA*^ the superior haplotype. Nevertheless, it remains unclear whether this 30‐bp deletion in *OsLHT5* enhances the capacity and affinity of amino acid transport. Previous studies have demonstrated that OsLHT1 can transport neutral and acidic amino acids; as a member of the same family, it remains to be investigated whether *OsLHT5* influences rice tillering via a specific amino acid, and whether it modulates salt tolerance through alternative pathways.

Amino acid transporters can modulate grain size and acid content (Peng et al. 2014; Shi et al. 2023). Given that *OsLHT5* exhibits relatively high expression in post‐flowering panicles (Figure S3c), it may potentially influence grain size and acid content. To explore this, we examined the effects of *LHT5*‐OE lines on grain size and amino acid content under HN and LN conditions (Figure S7a). Our results revealed that, compared with the wild type, *LHT5* ^*HapA*^‐OE lines showed significantly increased grain length and thousand‐grain weight, accompanied by a significant decrease in amino acid content. In contrast, *LHT5* ^*HapB*^‐OE lines exhibited significantly reduced grain length and thousand‐grain weight, with a concurrent significant decrease in amino acid content (Figure S7b–g). These findings suggest that different haplotypes of *OsLHT5* regulate amino acid transport during the grain‐filling stage, thereby influencing grain development.

In summary, our findings reveal that *OsLHT5* positively regulates NUE and salt tolerance in rice. Field trials conducted under both high‐ and low‐nitrogen conditions confirmed that the superior haplotype of *OsLHT5* significantly enhances grain yield. We further demonstrated that under low‐nitrogen conditions and salt stress, the superior haplotype of *OsLHT5* promotes the accumulation of specific amino acids, including glutamate and proline. These results not only establish a novel mechanism underlying NUE and stress tolerance but also provide a theoretical basis for the development of innovative rice breeding strategies, which may facilitate the improvement of stress‐tolerant crop varieties and contribute to sustainable food security.

## 4 Materials and Methods

### 4.1 Plant Materials and Field Experiments

The seeds of the 175 accessions were collected, stored and supplied by the State Key Laboratory of Crop Genetics & Germplasm Enhancement and Utilisation, Jiangsu Collaborative Innovation Center for Modern Crop Production, Nanjing Agricultural University, China. Germinate, transplant, and cultivate the 175 cultivars simultaneously in the same fields (HN or LN). The 175 rice cultivars could grow productive tillers with normal grains for harvest in Nanjing, China (31°139′ N, 119°22′ E, 30 m above sea level). All materials were planted in the field at the experimental farm of Nanjing Agricultural University, Nanjing, China (31°139′ N, 119°22′ E, 30 m above sea level). For the field experiments, the accessions were grown in a completely randomised block design with four replicates. The field experiments were carried out as a randomised block design with two nitrogen levels (with 300 kg/ha net nitrogen and 0 kg/ha net nitrogen) in two blocks. Phosphate and potassium fertilisers were both applied at 100 kg/ha. There were 20 cm and 17 cm between rows and individuals, respectively. Rice seedlings were cultured in IRRI nutrient solution (Zhang et al. 2021). All transgenic materials were obtained through *Agrobacterium tumefaciens* ‐mediated transformation as previously described (Hiei et al. 1994). Daytime conditions in the greenhouse were 30°C for 14 h; night‐time conditions were 28°C and dark for 10 h.

### 4.2 Genome‐Wide Association Study (GWAS)

We investigated plant height and the number of productive tillers at the mature stage under low nitrogen (LN) and high nitrogen (HN) conditions. The ratio of plant height under LN to HN (PHR) and effective panicle number under LN to HN (EPNR) were calculated and used as a proxy for nitrogen use efficiency (NUE) in the subsequent genome‐wide association study (GWAS). GWAS was conducted, and candidate genes were prioritised following procedures described in our previously published work, with minor modifications (Wu et al. 2024).

### 4.3 Transgenic Lines Construction

To generate transgenic rice of *OsLHT5*, Os*LHT5* coding sequences were amplified by PCR using cDNA from different haplotype cultivar templates and cloned into the pMD19‐T vector (Code. No. 6013, Takara) for subsequent vector construction. KOD‐FX (Code No. KFX‐101, TOYOBO CO. LTD, Japan) DNA polymerase was used for PCR amplification. Primer sequences are listed in Table S1.

To construct vectors for *OsLHT5*‐overexpression, *LHT5* ^*HapA*^, *LHT5* ^*HapB*^ and *LHT5* ^*HapC*^ coding sequences were used as templates for PCR. Primers containing *Hind*III and *Bam*HI restriction sites were used to insert PCR products into the pCUbi1390 vector by homologous recombination and were used for transformation of cultivar Nipponbare.

To construct *OsLHT5* loss‐of‐function mutants, rice gene editing was performed by CRISPR–Cas9 and single‐guide RNAs were designed with CRISPR‐P v2.0 (http://crispr.hzau.edu.cn/CRISPR2/). Constructs for the gene editing were generated using a CRISPR plasmid toolbox as described previously (Liu et al. 2017). The primers used for editing are listed in Table S1.

Sequence‐confirmed recombination plasmids were transformed into *A. tumefaciens* EHA105 for transformation of callus as described previously (Hiei et al. 1994). Transformed plants were identified by selection on 50 mg/L hygromycin B (CAS: 31282–04‐9, Solarbio Science & Technology Co. Ltd., Beijing, China) and PCR detection of gene recombinant vectors. Homozygous, Cas9‐free mutants were obtained through genetic segregation and genotyping by PCR and Sanger sequencing. Transgenic overexpression plants were subsequently analysed by qRT–PCR for gene‐expression levels. Primer sequences are listed in Table S1.

### 4.4 Quantitative Real‐Time PCR (qRT–PCR)

Total RNA was extracted and purified using RNA purification reagent (Code No. DP432, TIANGEN). cDNA synthesis was performed with 2 μg RNA using Prime Script Reverse Transcriptase and Oligo (dT) primers (TaKaRa Code: D2680A). Real‐time PCR was done in a Real Time PCR machine (I‐Cycle, BioRad) according to the manufacturer's manuals in a reaction mixture of 20 μL of TB Green Fast qPCR Mix (CellAmp Direct TB Green qRT‐PCR Kit). Amplification conditions were as previously described (Tang et al. 2019). *ACTIN1* (*LOC_Os03g13170*) was used as a reference gene, and three biological replicates were analysed. $2^{-\Delta \Delta C_{t}}$ was used to calculate relative gene expression (Yu et al. 2023). Primer sequences are listed in Table S1.

### 4.5 Quantification of Total N Contents

Plants were grown hydroponically for 14 days with either 2 or 0.2 mM NH_4_NO_3_. Harvested plants were washed with ddH_2_O and separated, then placed in an oven at 105°C for 30 min to inactivate enzymes and finally dried to a constant weight at 70°C for 7 days. Total N concentration was determined by Dumas Nitrogen Analyser (VELP scientific, NDA 702).

### 4.6 Amino Acid Analysis

Plants were grown hydroponically for 14 days with either 2 or 0.2 mM NH_4_NO_3_ treatment and shoots were separated for free amino acid analysis. All shoots were collected in liquid nitrogen, then mixed by mortar. The samples were then transferred to 10 mL centrifuge tubes, frozen in liquid nitrogen, and then transferred to a freeze dryer (BILON, FD‐2C, Shanghai, China) for 3 days. Accurately weigh 30 mg of dry sample in a mortar and add 1 mL of 80% ethanol and grind until volatile. The sample was washed with 3 mL of 4% sulfosalicylic acid three times into a 10 mL centrifuge tube. Extraction was carried out by ultrasonic shaking for 20 min. centrifugation was performed at 5000 r/min for 25 min. The supernatant was transferred to a 10 mL volumetric flask. Add 3 mL of sulfosalicylic acid to the precipitate and repeat the extraction twice, combine the three times of the supernatant, ultrapure water was fixed. The supernatant was filtered through 0.22 mm filter head and ready for sampling. Free amino acid contents were determined by Ultra HighSpeed Automatic Amino Acid Analyser LA8080 (Xie et al. 2023).

### 4.7 Protoplast Isolation and Subcellular Localisation

*OsLHT5* cDNA amplified from wild type cultivar Nipponbare was cloned into the pAN580 vector (Duan et al. 2019) (linearised with *Xba*I and *Bam*HI) between the cauliflower mosaic virus *35S* promoter and the green fluorescent protein (GFP) protein sequence to form a translational fusion with the N‐terminus of GFP. OsSCAMP1–mCherry was used as a plasmamembrane marker as previously reported (Cai et al. 2012). GFP fluorescence was observed using a confocal laser‐scanning microscope (Leica TCS SP5; Leica Microsystems, Wetzlar, Germany) using 488 nm excitation and 520 nm emission. mCherry fluorescence was observed using 550 nm excitation and 610 nm emission.

### 4.8 Quantification and Statistical Analysis

All the results were analysed and plotted in GraphPad Prism v.9 (www.graphpad.com/features). Significant differences between the two sets of data were determined by Student's *t*‐test (*p < 0.05, **p < 0.01, ***p < 0.001, ****p < 0.0001; ns: *p* > 0.05), whereas differences among more than two sets of data were analysed with one‐way analysis of variance (ANOVA) followed by Duncan's multiple comparisons (*p* < 0.05). The sample size used to derive the statistics is indicated in the figure legends or in the figures.

## Author Contributions

C.W. and J.W. directed the project. S.W. and X.J. performed the experiments. C.W. and G.C. conceived and designed the research. W.W., C.X., M.D., S.X., X.C. and Y.T. participated in the experiments. C.W., S.W., X.J., G.C. and J.T. wrote and finalised the manuscript. All authors approved the manuscript.

## Conflicts of Interest

The authors declare no conflicts of interest.

## Supporting information


**Appendix S1:** pbi70584‐sup‐0001‐AppendixS1.docx.

**Appendix S2:** pbi70584‐sup‐0002‐AppendixS2.zip.

## Data Availability

The data that support the findings of this study are available in Appendices S1 and S2 of this article.
