# Supplementary material for: An Elite Haplotype of Nitrogen‐Use‐Efficiency Gene LHT5 Enhances Salt Tolerance in Rice
Source: Plant Biotechnol J. 2026 Feb 6;24(6):3514–27. doi: 10.1111/pbi.70584 (PMC13205599; doi:10.1111/pbi.70584)
Supplement: Supplementary file 1 — Appendix S1: pbi70584‐sup‐0001‐AppendixS1.docx. [file PBI-24-3514-s002.docx]

**Supplementary figures**


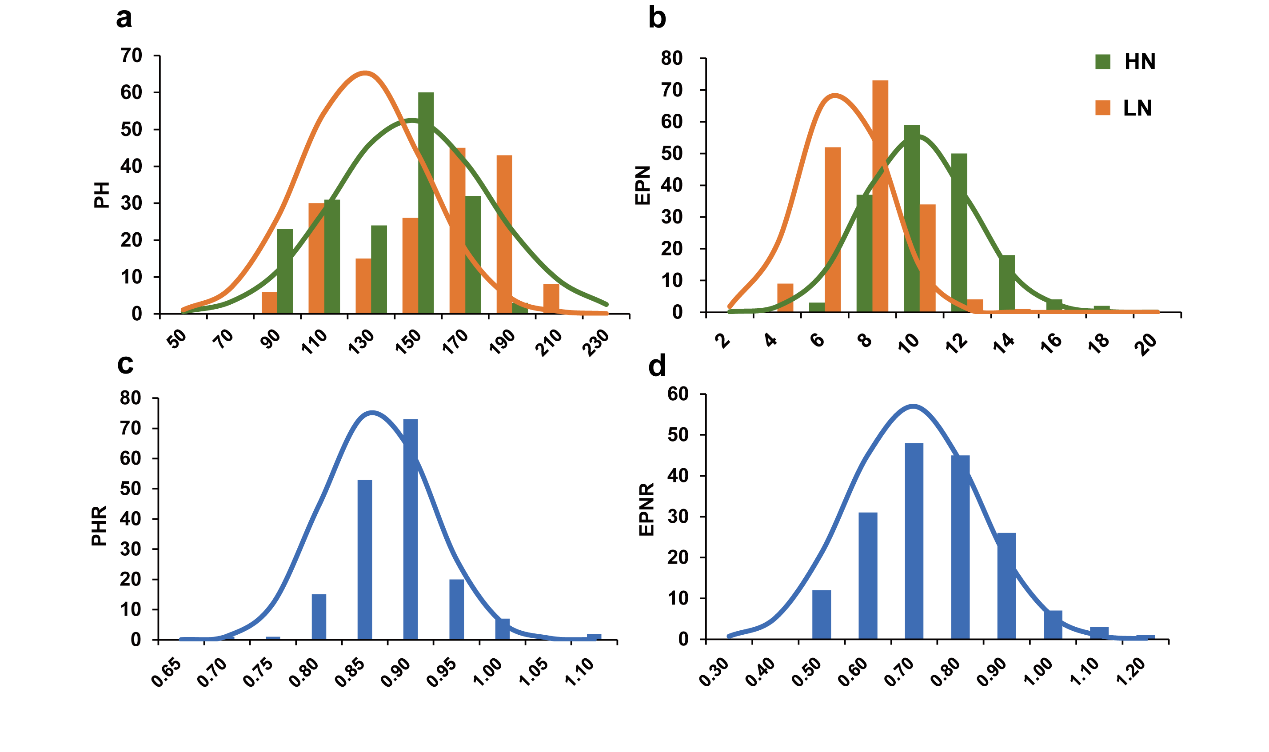


**Supplementary figure 1**

a-b. Frequency distributions for EPN and PH of the 175 rice varieties measured under LN and HN fields.

c-d. Frequency distributions for EPNR and PHR of the 175 rice varieties.


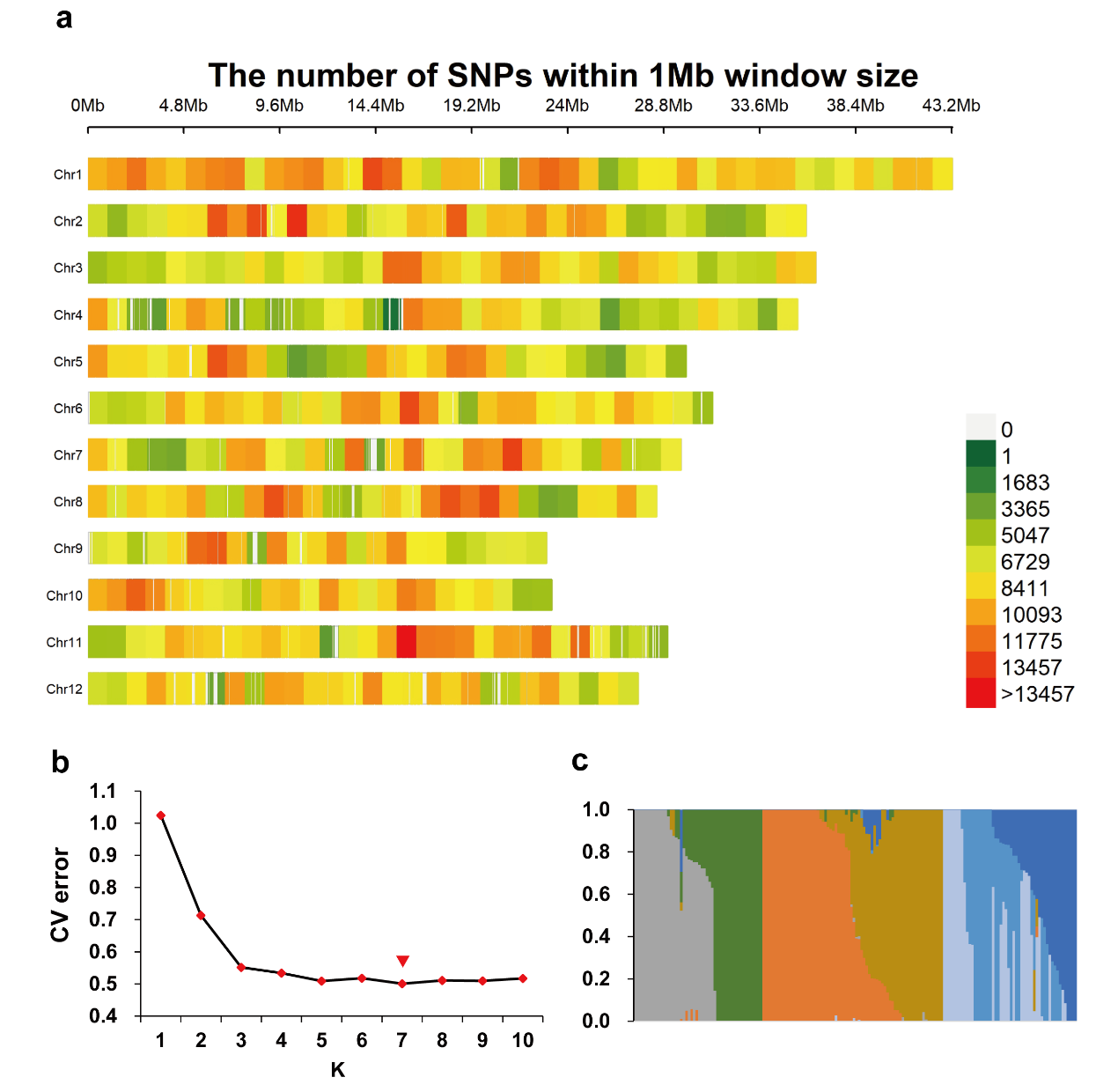


**Supplementary figure 2.** **SNPs density and population structure analysis.**

1. The distribution density of SNPs on each chromosome.
2. The CV error value was much lower for the model parameter K = 7 than for other values of K*.*
3. Posterior probabilities of each rice variety belonging to seven subpopulations calculated by ADMIXTURE software. Each accession is represented by a vertical bar. The colored subsections within each vertical bar indicate membership coefficient (Q) of the accession to different clusters.


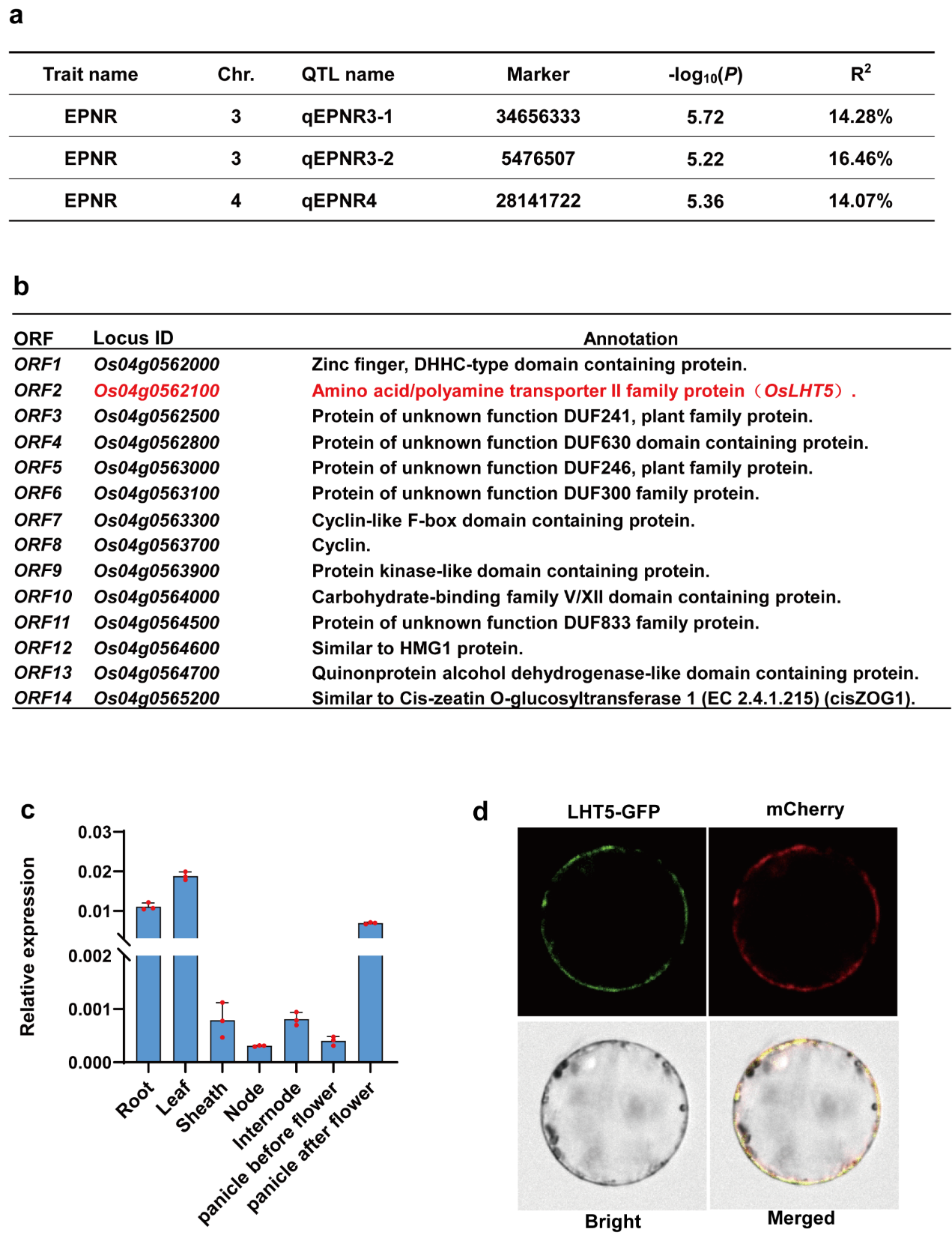


**Supplementary figure 3.** **Functional analysis of *OsLHT5***

1. Three QTLs associated with EPNR.
2. Gene annotation of fourteen *ORFs* located in the LD on Chr. 4.
3. Relative expression levels of *OsLHT5* in various tissues of the wild-type cultivar Nipponbare, as determined by qRT-PCR. *ACTIN1* was used as the internal reference gene for normalization. Bars represent the mean ± SD of *n* = 3.
4. Confocal microscopy analysis of OsLHT5–GFP subcellular localization in rice protoplasts. OsSCAMP1–mCherry was used as plasma-membrane marker. Scale bar = 5 μm.


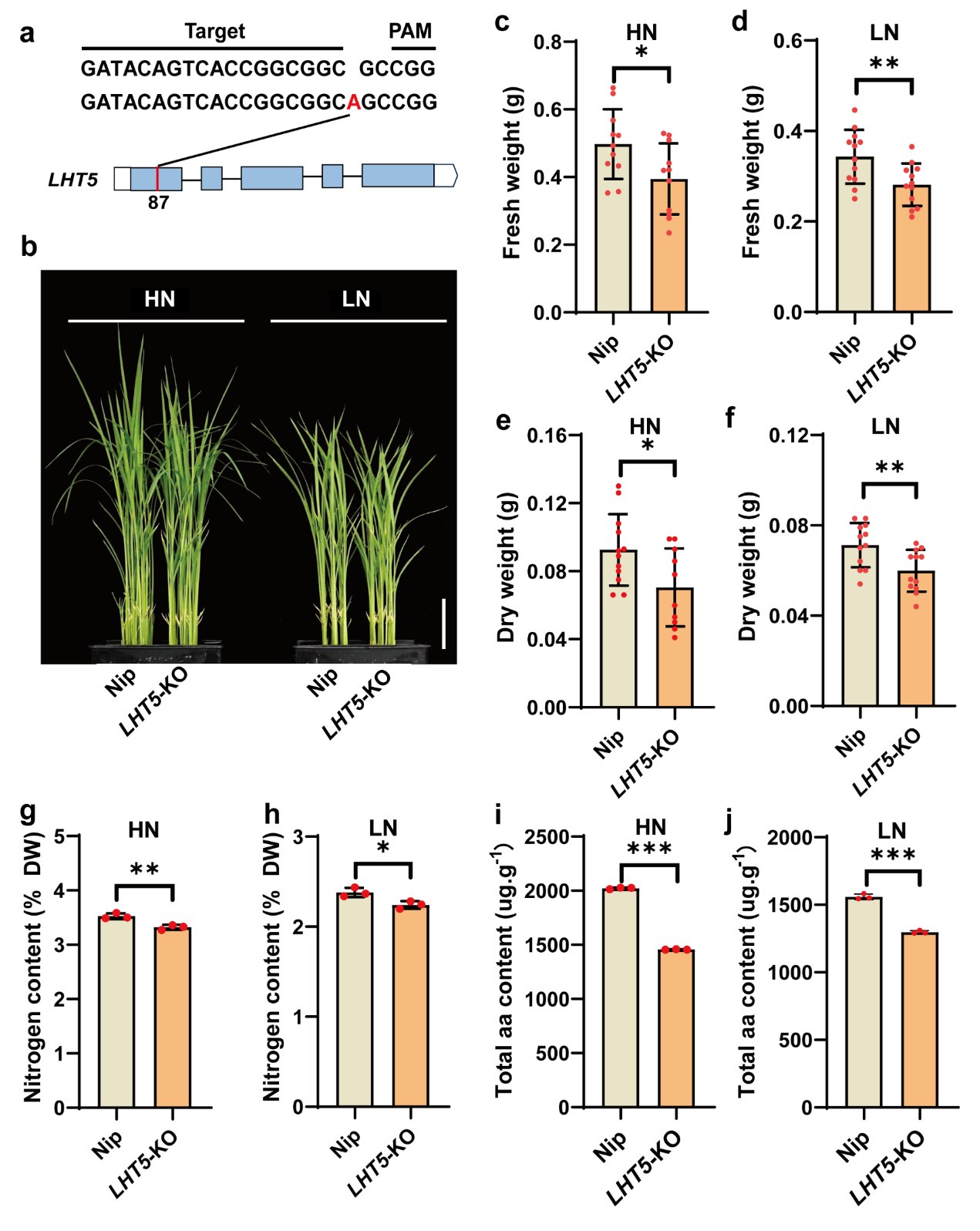


**Supplementary figure 4.** **Phenotypes of *LHT5-*KO mutant at seeding stages.**

1. Schematic representation of CRISPR–Cas9-mediated gene editing in the *OsLHT5* locus. The blue box indicates the *OsLHT5* amino acid sequence. A 1-bp insertion was identified in the *LHT5*-KO mutant.
2. Phenotypes of Nipponbare and *LHT5-*KO mutant under HN and LN treatment at seeding stages.
3. Phenotypic analysis of fresh weight, dry weight, nitrogen content and total amino acid content of wild-type cultivar Nipponbare and *LHT5-*KO mutant grown in HN (2 mM) and LN (0.2 mM) NH_4_NO_3_ conditions at 14-d old. Bars represent the mean ± SD. (c-f) *n* = 11, (g-j) *n* = 3.

Statistically significant differences are indicated by different letters. *, **, and ***, **** of *t*-test indicate significant differences at *P*<0.05, *P*<0.01, and *P*<0.001, *P*<0.0001.


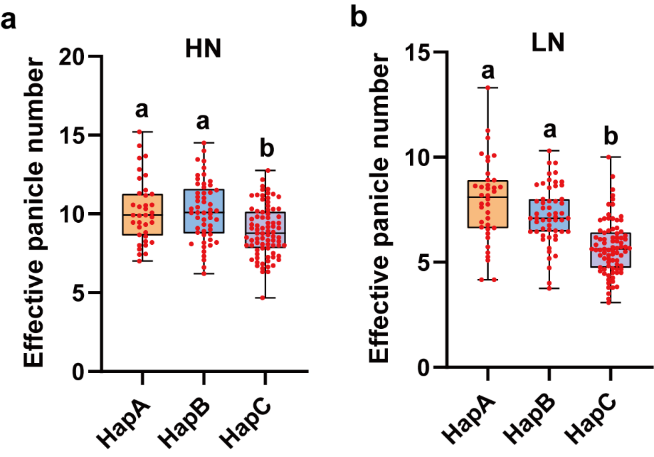


**Supplementary figure 5. Comparison of EPN in three haplotypes of OsLHT5.**

a-b. Effective panicle number of rice populations under high and low nitrogen conditions in paddy fields.


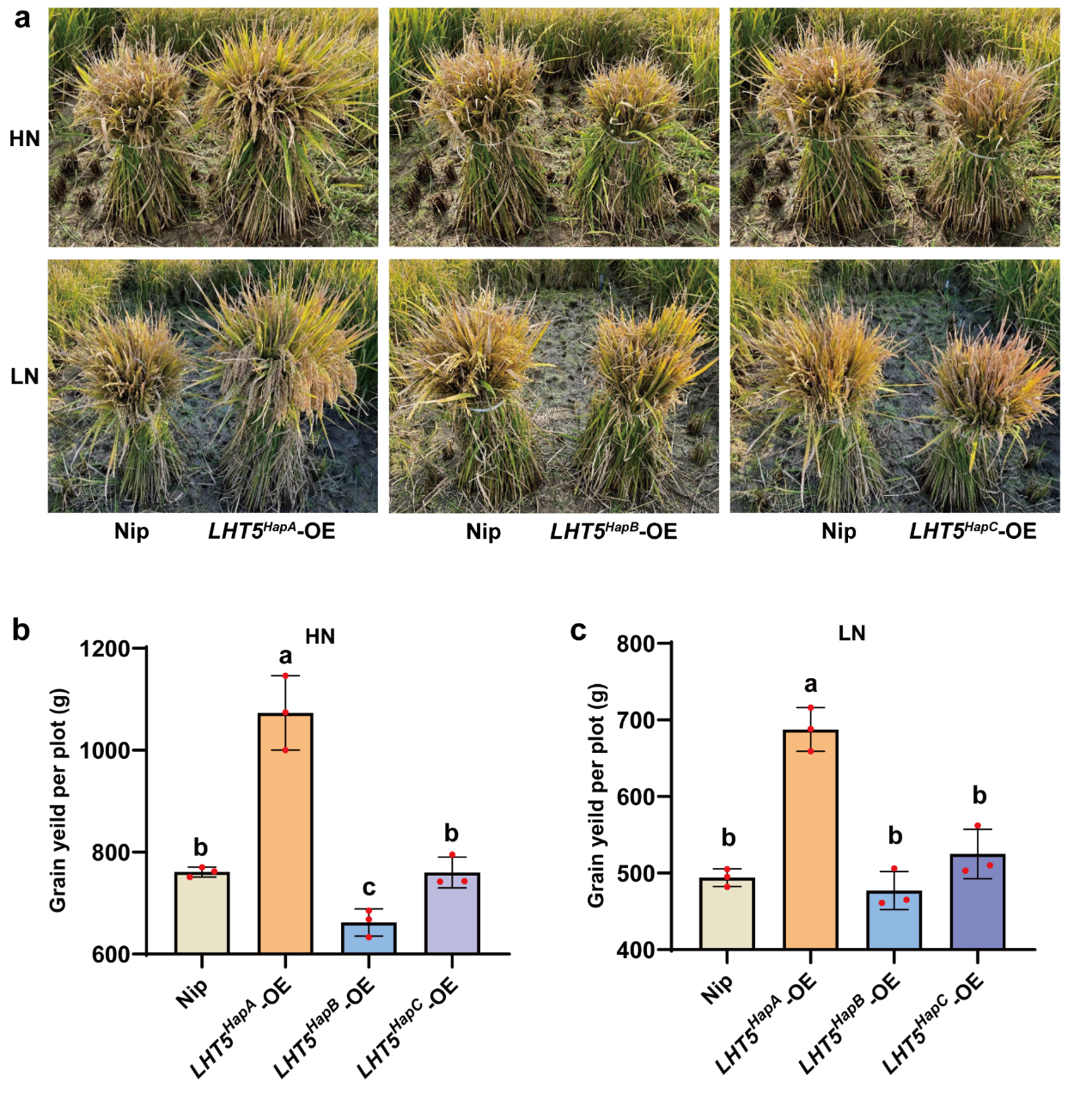


**Supplementary figure 6.** **Phenotypes of *LHT5*-OE and knockout lines at mature stages.**

1. Phenotypes of Nipponbare, *LHT5*-OE and knockout lines grown in HN and LN paddy fields at mature stages.

b-c. Phenotypic analysis of grain yeild per plot (8 plants × 4 rows) of Nipponbare, *LHT5-*OE in HN and LN paddy fields. Bars represent the mean ± SD. *n* = 3.

Statistical significance was calculated by one-way ANOVA with Duncan’s multiple range test (*P* < 0.05). *, **, and ***, **** of *t*-test indicate significant differences at *P*<0.05, *P*<0.01, and *P*<0.001, *P*<0.0001. Statistically significant differences are indicated by different letters.


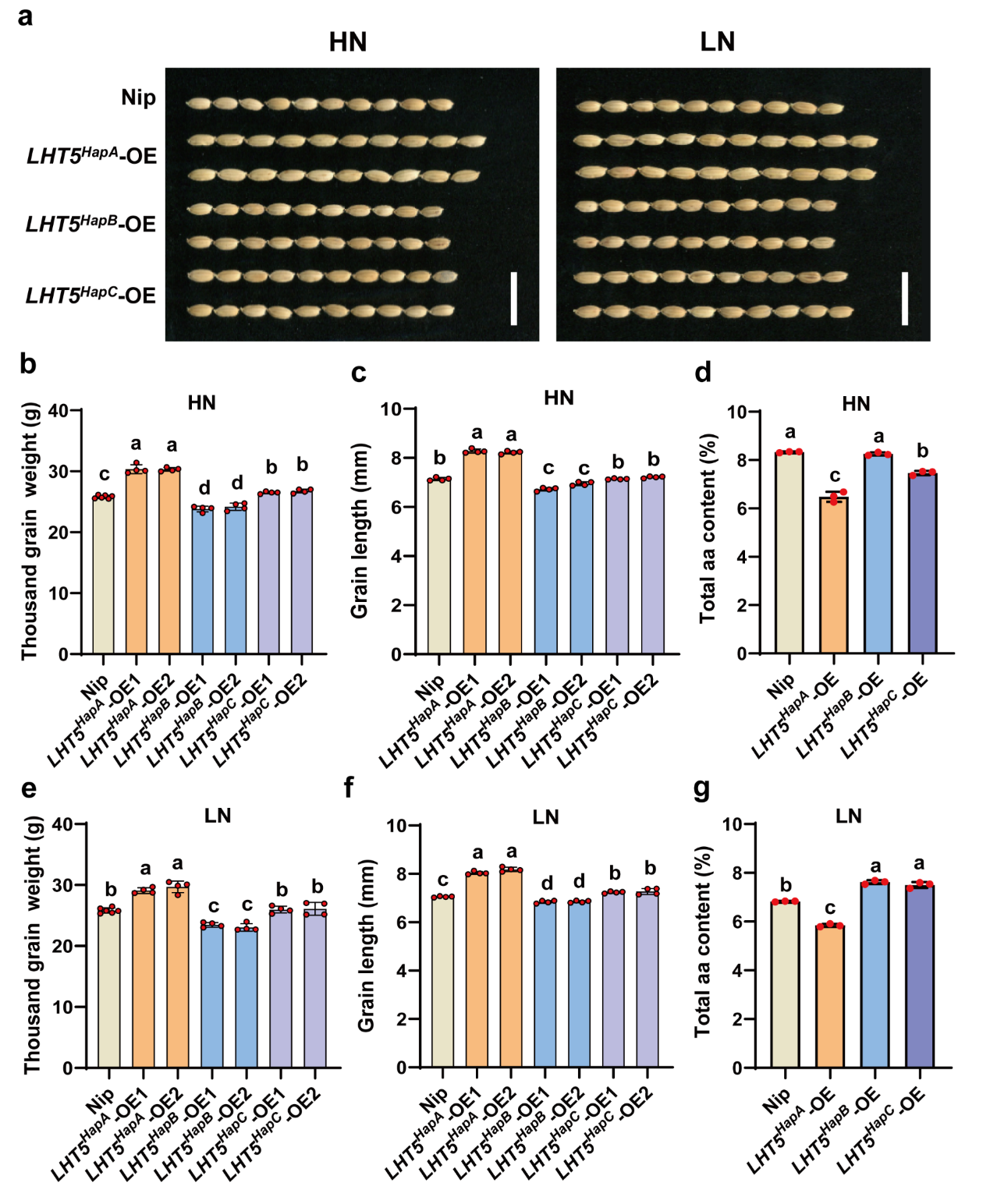


**Supplementary figure 7.** **Grain size and amino acid content of *LHT5*-OE lines**

1. Phenotypes of Nipponbare and *LHT5*-OE lines grown in HN and LN paddy fields at mature stages.
2. Phenotypic analysis of the grain length, thousand-grain weight and amino acid content of Nipponbare and *LHT5-*OE lines in HN and LN paddy fields. Bars represent the mean ± SD. *n* ≥ 3

Statistical significance was calculated by one-way ANOVA with Duncan’s multiple range test (*P* < 0.05). Statistically significant differences are indicated by different letters.

**Table S1. Primers used in this study.**

| **Primer name** | **Primer sequence 5’ - 3’** |
| --- | --- |
| LHT5-CDS-F | ATGTCGAGCGAGGTGACGTC |
| LHT5-CDS-R | TCACTGGAAATCCGCAGGCT |
| LHT5-CRISPR-F | GGCAGATACAGTCACCGGCGGCGC |
| LHT5-CRISPR-R | AAACGCGCCGCCGGTGACTGTATC |
| pAN580- LHT5-GFP-F | CGGAGCTAGCTCTAGAATGTCGAGCGAGGTGACGTC |
| pAN580- LHT5-GFP-R | TGCTCACCATGGATCCCTGGAAATCCGCAGGCTTGA |
| 1390- LHT5-F | TTACTTCTGCACTAGGTACCATGTCGAGCGAGGTGACGTCGG |
| 1390- LHT5-R | GAATTCCCGGGGATCC TCACTGGAAATCCGCAGGCT |
| qRT- LHT5-F | CATCATCGCACTCTGCTTGTACC |
| qRT- LHT5-R | GATCCCATTTGGTGGTATCTGGTC |
| LHT5-CRISPR-JC-F | CCCTCACACCTACGCTCCAT |
| LHT5-CRISPR-JC-R | GGCGACGGGGAGCACGAGCGCC |
| qRT- P5CS1-F | TTGGATTGGGTGCTGAGGTTGG |
| qRT- P5CS1-R | ACGACATCCTTGTCACCATTCACC |
| qRT- P5CS2-F | TTCCCAGATCAAAGCAGCAACC |
| qRT- P5CS2-R | AAACATGGCAGATACCGTCAGC |
