# Supplementary material for: An Elite Haplotype of Nitrogen‐Use‐Efficiency Gene LHT5 Enhances Salt Tolerance in Rice
Source: Plant Biotechnol J. 2026 Feb 6;24(6):3514–27. doi: 10.1111/pbi.70584 (PMC13205599; doi:10.1111/pbi.70584)
Supplement: Supplementary file 2 — Appendix S2: pbi70584‐sup‐0002‐AppendixS2.zip. [file PBI-24-3514-s001.zip › Supplementary Material for question 4 of review2/LHT5-HapC.html]

LHT5-C | Report


|  |  |  |
| --- | --- | --- |
|  |  | SWISS-MODEL Homology Modelling Report |

## Model Building Report

This document lists the results for the homology modelling project "LHT5-C" submitted to SWISS-MODEL workspace
on Aug. 23, 2023, 2:17 p.m..The submitted primary amino acid sequence is given in Table T1.

If you use any results in your research, please cite the relevant publications:

- Waterhouse, A., Bertoni, M., Bienert, S., Studer, G., Tauriello, G., Gumienny, R.,
  Heer, F.T., de Beer, T.A.P., Rempfer, C., Bordoli, L., Lepore, R., Schwede, T.
  SWISS-MODEL: homology modelling of protein structures and complexes.
  Nucleic Acids Res. 46(W1), W296-W303 (2018).
- Bienert, S., Waterhouse, A., de Beer, T.A.P., Tauriello, G., Studer,
  G., Bordoli, L., Schwede, T. The SWISS-MODEL Repository - new features and
  functionality. Nucleic Acids Res. 45, D313-D319 (2017).
- Studer, G., Tauriello, G., Bienert, S.,
  Biasini, M., Johner, N., Schwede, T. ProMod3 - A versatile homology
  modelling toolbox. PLOS Comp. Biol. 17(1), e1008667 (2021).
- Studer, G., Rempfer, C., Waterhouse, A.M.,
  Gumienny, G., Haas, J., Schwede, T. QMEANDisCo - distance constraints
  applied on model quality estimation. Bioinformatics 36, 1765-1771 (2020).
- Bertoni, M., Kiefer, F., Biasini, M., Bordoli, L.,
  Schwede, T. Modeling protein quaternary structure of homo- and
  hetero-oligomers beyond binary interactions by homology. Scientific
  Reports 7 (2017).

## Results

The SWISS-MODEL template library (SMTL version 2023-08-16, PDB release 2023-08-11) was searched with
for evolutionary related structures matching the target sequence in Table T1. For details on the template search, see Materials and Methods. Overall 142 templates were found (Table T2).

## Models

The following models were built (see Materials and Methods "Model Building"):

| Model #01 | File | Built with | Oligo-State | Ligands | GMQE |
| --- | --- | --- | --- | --- | --- |
|  | PDB | ProMod3 3.3.0 | monomer | None | 0.85 |

|  |  |  |
| --- | --- | --- |
|  |  |  |

| Template | Seq Identity | Oligo-state | QSQE | Found by | Method | Resolution | Seq Similarity | Range | Coverage | Description |
| --- | --- | --- | --- | --- | --- | --- | --- | --- | --- | --- |
| A0A1D6E3A9.1.A | 84.28 | monomer | - | AFDB search | AlphaFold v2 | - | 0.57 | 3 - 512 | 0.99 | Lysine histidine transporter-like 7 |

  

### The template contained no ligands.

  

```
Target    MSSEVTSVPPTPTPPPVSTPPSQIQSPA---AP----ASS-R--ASPLRGMGTPNIASPVRKAVASVSGYLGEVGQMTRL  
A0A1D6E3A9.1.A--SEVESAQPSPRPPPVSTPPSQIHSPAPGRSPLHAMASPLRAVASPLRAMATP-LASPVRKAVAGVRECLEEVGHITRL  
  
Target    ADPRDAWLPITESRSGNAYYAAFHSLSSGIGFQALVLPVAFSLLGWTWAIICLTVAFAWQLYTLWLLVKLHEPVAGGTRY  
A0A1D6E3A9.1.AADPRDAWLPITESRSGNAYYAAFHNLSSGIGFQALVLPTAFASLGWTWAIICLTLAFGWQLYTLWLLVRLHEPVAGATRY  
  
Target    SRYMYLATTVFGEKWGKILALLPVMYLSAGTCTALIIVGGGSMKLLFNIACGEVCLARPLTTVEWYLVFVCVAALLSQLP  
A0A1D6E3A9.1.ASRYMHLATTVFGERWAKILALLPVMYLSAGICTALIIVGGGSMKLLFGIACGEPCPARPPTTVEWYLVFVCAAVLLSQLP  
  
Target    NLNSIAGVSLVGATAAVAYCTMIWVVSVAKGRVAGVSYDPVRTTDEEDGAIGILNGLGIIAFAFRGHNLVLEIQATMPST  
A0A1D6E3A9.1.ANLNSIAGVSLVGATAAVAYCTMIWTVSVAKGRVPAVSYDPVKAPSDVDGALAILNGLGIIAFAFRGHNVVLEIQGTMPST  
  
Target    LKHPSHVPMWKGVKAAYVIIALCLYPVAVGGFWAYGDQIPPNGILSALYKFHSQDVSRVVLGTATLLVIVNCLTTYQIYA  
A0A1D6E3A9.1.ALKHPSHVPMWKGVKVAYAIIALCLYPVAIGGFWAYGNQIPPNGILSALYKFHSRDTSRLVLGVTTTLVIVNCLTTFQIYA  
  
Target    MPVFDNMETGYVHKKNRPCPWWMRAGFRALFGAINLLIAVALPFLSELAGLLGGISLPVTLAYPCFMWVAIMRPAKGTAM  
A0A1D6E3A9.1.AMPVYDNMEAGYVHKKNRPCPWWMRSGFRAFFGAVNFLIAVALPFLSQLAGLLGGISLPVTLAYPCFMWVAIKKPRKGTAT  
  
Target    WYTNWGLGSLGMGLSFVLIVGNLWGLVEKGLHVKFFKPADFQ  
A0A1D6E3A9.1.AWNVNWALGILGMSISLVLIVGNLWGLVEKGLRVKFFKPADSQ
```

  


---

  

| Model #02 | File | Built with | Oligo-State | Ligands | GMQE | QMEANDisCo Global |
| --- | --- | --- | --- | --- | --- | --- |
|  | PDB | ProMod3 3.3.0 | monomer | None | 0.43 | 0.48 ± 0.05 |

|  |  |  |
| --- | --- | --- |
|  |  |  |

| Template | Seq Identity | Oligo-state | QSQE | Found by | Method | Resolution | Seq Similarity | Range | Coverage | Description |
| --- | --- | --- | --- | --- | --- | --- | --- | --- | --- | --- |
| 6c08.2.C | 13.45 | monomer | 0.00 | HHblits | X-ray | 3.17Å | 0.27 | 89 - 492 | 0.77 | Sodium-coupled neutral amino acid transporter 9 |

  

### Excluded ligands

| Ligand Name.Number | Reason for Exclusion | Description |
| --- | --- | --- |
| ARG.1 | Binding site not conserved. | ARGININE |

  

```
Target    MSSEVTSVPPTPTPPPVSTPPSQIQSPAAPASSRASPLRGMGTPNIASPVRKAVASVSGYLGEVGQMTRLADPRDAWLPI  
6c08.2.C  --------------------------------------------------------------------------------  
  
Target    TESRSGNAYYAAFHSLSSGIGFQALVLPVAFSLLGWTWAIICLTVAFAWQLYTLWLLVKLHEPVA-GGTRYSRYMYLATT  
6c08.2.C  DSPIKNPSIVTIFAIWNTMMGTSILSIPWGIKQAGFTLGIIIIVLMGLLTLYCCYRVLKSTKSIPYVDTSDWEFPDVCKY  
  
Target    VFGEKWGKILALLPVMYLSAGTCTALIIVGGGSMKLLFNIACGE------------------VC----------------  
6c08.2.C  YFGGFGKWSS-LVFSLVSLIGAMVVYWVLMSNFLFNTGKFIFNYVHNVQTSDAFGTQGTERVICPYPDVDPHGQSSTSLY  
  
Target    ----LARPLTTVE-------WYLVFVCVAALLSQLPNLNSIAGVSLVGATAAVAYCTMIWVVSVAKGRVAGVSYDPVRTT  
6c08.2.C  SGSDQSTGLEFDHWWSKTNTIPFYLILLLLPLLNFRSASFFARFTFLGTISVIYLIFLVTYKAIQLGFHLEFHWFDSSMF  
  
Target    DEEDGAIGILNGLGIIAFAFRGHNLVLEIQATMPSTLKHPSHVPMWKGVKAAYVIIALCLYPVAVGGFWAYGDQ------  
6c08.2.C  FVPEFRTLFPQLSGVLTLAFFIHNCIITLMKNN----KHQEN--NVRDLSLAYLLVGLTYLYVGVLIFAAFPSPPLSKEC  
  
Target    IPPNGILSALYKFHSQDVSRVVLGTATLLVIVNCLTTYQIYAMPVFDNMETGYVHKKNRPCPWWMRAGFRALFGAINLLI  
6c08.2.C  IEPN-FLDNFPS---S---DILVFVARTFLLFQMTTVYPLLGYLVRVQLMGQIFGNH--YPGFLHVFVLNVFVVGAGVLM  
  
Target    AVALPFLSELAGLLGGIS-LPVTLAYPCFMWVAIMRPAKGTAMWYTNWGLGSLGMGLSFVLIVGNLWGLVEKGLHVKFFK  
6c08.2.C  ARFYPNIGSIIRYSGALCGLALVFVLPSLIHMVSLKRRGEL--RWTSTLFHGFLILLGVANLLGQ---------------  
  
Target    PADFQ  
6c08.2.C  -----
```

  


---

  

| Model #03 | File | Built with | Oligo-State | Ligands | GMQE | QMEANDisCo Global |
| --- | --- | --- | --- | --- | --- | --- |
|  | PDB | ProMod3 3.3.0 | monomer | None | 0.23 | 0.46 ± 0.05 |

|  |  |  |
| --- | --- | --- |
|  |  |  |

| Template | Seq Identity | Oligo-state | QSQE | Found by | Method | Resolution | Seq Similarity | Range | Coverage | Description |
| --- | --- | --- | --- | --- | --- | --- | --- | --- | --- | --- |
| 5oqt.1.A | 13.04 | monomer | 0.00 | HHblits | X-ray | 2.86Å | 0.26 | 81 - 348 | 0.49 | Amino acid transporter |

  

### Excluded ligands

| Ligand Name.Number | Reason for Exclusion | Description |
| --- | --- | --- |
| ACT.13 | Not biologically relevant. | ACETATE ION |
| ALA.15 | Binding site not conserved. | ALANINE |
| CL.14 | Not biologically relevant. | CHLORIDE ION |
| CLR.16 | Binding site not conserved. | CHOLESTEROL |
| OLC.1 | Binding site not conserved. | (2R)-2,3-dihydroxypropyl (9Z)-octadec-9-enoate |
| OLC.2 | Not in contact with model. | (2R)-2,3-dihydroxypropyl (9Z)-octadec-9-enoate |
| OLC.3 | Binding site not conserved. | (2R)-2,3-dihydroxypropyl (9Z)-octadec-9-enoate |
| OLC.4 | Binding site not conserved. | (2R)-2,3-dihydroxypropyl (9Z)-octadec-9-enoate |
| OLC.5 | Binding site not conserved. | (2R)-2,3-dihydroxypropyl (9Z)-octadec-9-enoate |
| OLC.6 | Binding site not conserved. | (2R)-2,3-dihydroxypropyl (9Z)-octadec-9-enoate |
| OLC.7 | Binding site not conserved. | (2R)-2,3-dihydroxypropyl (9Z)-octadec-9-enoate |
| OLC.8 | Binding site not conserved. | (2R)-2,3-dihydroxypropyl (9Z)-octadec-9-enoate |
| OLC.9 | Binding site not conserved. | (2R)-2,3-dihydroxypropyl (9Z)-octadec-9-enoate |
| OLC.10 | Not in contact with model. | (2R)-2,3-dihydroxypropyl (9Z)-octadec-9-enoate |
| OLC.11 | Not in contact with model. | (2R)-2,3-dihydroxypropyl (9Z)-octadec-9-enoate |
| OLC.12 | Binding site not conserved. | (2R)-2,3-dihydroxypropyl (9Z)-octadec-9-enoate |
| OLC.17 | Binding site not conserved. | (2R)-2,3-dihydroxypropyl (9Z)-octadec-9-enoate |

  

```
Target    MSSEVTSVPPTPTPPPVSTPPSQIQSPAAPASSRASPLRGMGTPNIASPVRKAVASVSGYLGEVGQMTRLADPRDAWLPI  
5oqt.1.A  --------------------------------------------------------------------------------  
  
Target    TESRSGNAYYAAFHSLSSGIGFQALVLPVAFS-LLGWTWAIICLTVAFAWQLYTLWLLVKLHEPVAGGTRYSRYMYLATT  
5oqt.1.A  SLRKELGAFDLTMLGIGAIIGTGIFVLTGVAAAEHAGPALVLSFILSGLACVFAALCYAEFASTVPVS---GSAYTYSYA  
  
Target    VFGEKWGKILALLPVMYLSAGTCTALIIVGGGSMKLLFNIACGEVC---LA-RPLTTVEWYLVFVCV---AALLSQLPNL  
5oqt.1.A  TFGELIAWILGWDLILE-YGVASSAVAVGWSGYFQGLLSGFGIELPKALTSAYDPAKGTFIDLPAIIIVLFITFLLNLGA  
  
Target    NSIAGVSLVGATAAVAYCTMIWVVSVAKGRVAGVSYDPVRTTDEEDGAIGILNGLGIIAFAFRGHNLVLEIQATMPSTLK  
5oqt.1.A  KKSARFNAVIVAIKVAVVLLFLAVGVWYVKPEN--WT----PFMPYGFSGVATGAATVFFAYIGFDAVSTAAEEV----R  
  
Target    HPSHVPMWKGVKAAYVIIALCLYPVAVGGFWAYGDQIPPNGILSALYKFHSQDVSRVVLGTATLLVIVNCLTTYQIYAMP  
5oqt.1.A  NPQR-DMPIGIIVSLLVCTLLYIAVSLVLTGIVPYE--------------------------------------------  
  
Target    VFDNMETGYVHKKNRPCPWWMRAGFRALFGAINLLIAVALPFLSELAGLLGGISLPVTLAYPCFMWVAIMRPAKGTAMWY  
5oqt.1.A  --------------------------------------------------------------------------------  
  
Target    TNWGLGSLGMGLSFVLIVGNLWGLVEKGLHVKFFKPADFQ  
5oqt.1.A  ----------------------------------------
```

  


---

  

## Materials and Methods

## Template Search

Template search with
has been performed against the SWISS-MODEL template library (SMTL, last update: 2023-08-16, last included PDB release: 2023-08-11).

## Template Selection

For each identified template, the template's quality has been predicted from features of the target-template alignment.
The templates with the highest quality have then been selected for model building.

## Model Building

Models are built based on the target-template alignment using ProMod3 (Studer et al.). Coordinates which are conserved between the target and the template are copied from the template to the model. Insertions and deletions are remodelled using a fragment library. Side chains are then rebuilt. Finally, the geometry of the resulting model is regularized by using a force field.

## Model Quality Estimation

The global and per-residue model quality has been assessed using the QMEAN scoring function (Studer et al.).

## Ligand Modelling

Ligands present in the template structure are transferred by homology to the model when the following criteria are met: (a) The ligands are annotated as biologically relevant in the template library, (b) the ligand is in contact with the model, (c) the ligand is not clashing with the protein, (d) the residues in contact with the ligand are conserved between the target and the template. If any of these four criteria is not satisfied, a certain ligand will not be included in the model. The model summary includes information on why and which ligand has not been included.

## Oligomeric State Conservation

The quaternary structure annotation of the template is used to model the target sequence in its oligomeric form. The method (Bertoni et al.) is based on a supervised machine learning algorithm, Support Vector Machines (SVM), which combines interface conservation, structural clustering, and other template features to provide a quaternary structure quality estimate (QSQE). The QSQE score is a number between 0 and 1, reflecting the expected accuracy of the interchain contacts for a model built based a given alignment and template. Higher numbers indicate higher reliability. This complements the GMQE score which estimates the accuracy of the tertiary structure of the resulting model.

## References

- **BLAST**  
  Camacho, C., Coulouris, G., Avagyan, V., Ma, N., Papadopoulos, J.,
  Bealer, K., Madden, T.L. BLAST+: architecture and applications. BMC
  Bioinformatics 10, 421-430 (2009).
- **HHblits**  
  Steinegger, M., Meier, M., Mirdita, M., Vöhringer, H., Haunsberger,
  S. J., Söding, J. HH-suite3 for fast remote homology detection and
  deep protein annotation. BMC Bioinformatics 20, 473 (2019).

## Table T1:

Primary amino acid sequence for which templates were searched and models were built.

MSSEVTSVPPTPTPPPVSTPPSQIQSPAAPASSRASPLRGMGTPNIASPVRKAVASVSGYLGEVGQMTRLADPRDAWLPITESRSGNAYYAAFHSLSSGI  
GFQALVLPVAFSLLGWTWAIICLTVAFAWQLYTLWLLVKLHEPVAGGTRYSRYMYLATTVFGEKWGKILALLPVMYLSAGTCTALIIVGGGSMKLLFNIA  
CGEVCLARPLTTVEWYLVFVCVAALLSQLPNLNSIAGVSLVGATAAVAYCTMIWVVSVAKGRVAGVSYDPVRTTDEEDGAIGILNGLGIIAFAFRGHNLV  
LEIQATMPSTLKHPSHVPMWKGVKAAYVIIALCLYPVAVGGFWAYGDQIPPNGILSALYKFHSQDVSRVVLGTATLLVIVNCLTTYQIYAMPVFDNMETG  
YVHKKNRPCPWWMRAGFRALFGAINLLIAVALPFLSELAGLLGGISLPVTLAYPCFMWVAIMRPAKGTAMWYTNWGLGSLGMGLSFVLIVGNLWGLVEKG  
LHVKFFKPADFQ

## Table T2:

| Template | Seq Identity | Oligo-state | QSQE | Found by | Method | Resolution | Seq Similarity | Coverage | Description |
| --- | --- | --- | --- | --- | --- | --- | --- | --- | --- |
| A0A1D6E3A9.1.A | 84.28 | monomer | - | AFDB search | AlphaFold v2 | NA | 0.57 | 0.99 | Lysine histidine transporter-like 7 |
| 6c08.2.C | 13.45 | monomer | - | HHblits | X-ray | 3.17Å | 0.27 | 0.77 | Sodium-coupled neutral amino acid transporter 9 |
| 7kgv.2.A | 14.29 | monomer | - | HHblits | X-ray | 3.40Å | 0.28 | 0.72 | Sodium-coupled neutral amino acid transporter 9 |
| 6c08.1.C | 13.45 | monomer | - | HHblits | X-ray | 3.17Å | 0.27 | 0.77 | Sodium-coupled neutral amino acid transporter 9 |
| 7kgv.1.A | 14.29 | monomer | - | HHblits | X-ray | 3.40Å | 0.28 | 0.72 | Sodium-coupled neutral amino acid transporter 9 |
| 6f34.1.A | 14.23 | monomer | - | HHblits | X-ray | 3.13Å | 0.26 | 0.49 | Amino acid transporter |
| 5j4i.1.A | 16.06 | homo-dimer | 0.07 | HHblits | X-ray | 2.21Å | 0.27 | 0.49 | Arginine/agmatine antiporter |
| 3ob6.1.A | 16.06 | homo-dimer | 0.05 | HHblits | X-ray | 3.00Å | 0.27 | 0.49 | AdiC arginine:agmatine antiporter |
| 7s1x.1.A | 12.50 | monomer | - | HHblits | EM | NA | 0.25 | 0.50 | Solute carrier family 12 member 2 |
| 3l1l.1.A | 15.60 | homo-dimer | 0.05 | HHblits | X-ray | 3.00Å | 0.26 | 0.49 | Arginine/agmatine antiporter |
| 7nf6.1.A | 9.06 | monomer | - | HHblits | EM | NA | 0.25 | 0.50 | B(0,+)-type amino acid transporter 1 |
| 6li9.1.B | 9.06 | monomer | - | HHblits | EM | NA | 0.25 | 0.50 | b(0,+)-type amino acid transporter 1 |
| 7zgo.1.A | 12.65 | monomer | - | HHblits | EM | NA | 0.25 | 0.49 | Solute carrier family 12 member 2 |
| 3gia.1.A | 8.50 | monomer | - | HHblits | X-ray | 2.32Å | 0.25 | 0.48 | Uncharacterized protein MJ0609 |
| 7s1y.1.A | 12.50 | monomer | - | HHblits | EM | NA | 0.25 | 0.50 | Solute carrier family 12 member 2 |
| 7smp.1.A | 13.04 | monomer | - | HHblits | EM | NA | 0.25 | 0.49 | Solute carrier family 12 member 2 |
| 6f2g.1.A | 12.45 | monomer | - | HHblits | X-ray | 2.92Å | 0.26 | 0.49 | Putative amino acid/polyamine transport protein |
| 3gi8.1.A | 9.31 | monomer | - | HHblits | X-ray | 2.59Å | 0.25 | 0.48 | Uncharacterized protein MJ0609 |
| 7y6i.1.A | 13.10 | monomer | - | HHblits | EM | NA | 0.25 | 0.49 | Solute carrier family 12 member 3 |
| 3ncy.1.A | 15.60 | homo-dimer | 0.10 | HHblits | X-ray | 3.20Å | 0.27 | 0.49 | AdiC |
| 7p9v.1.B | 11.76 | monomer | - | HHblits | EM | NA | 0.26 | 0.50 | Cystine/glutamate transporter |
| 7p9u.1.B | 11.76 | monomer | - | HHblits | EM | NA | 0.26 | 0.50 | Cystine/glutamate transporter |
| 7epz.1.B | 11.76 | monomer | - | HHblits | EM | NA | 0.26 | 0.50 | Cystine/glutamate transporter |
| 5oqt.1.A | 13.04 | monomer | - | HHblits | X-ray | 2.86Å | 0.26 | 0.49 | Amino acid transporter |
| 7smp.1.B | 13.04 | monomer | - | HHblits | EM | NA | 0.25 | 0.49 | Solute carrier family 12 member 2 |
| 6npk.1.A | 12.25 | monomer | - | HHblits | EM | NA | 0.25 | 0.49 | Solute carrier family 12 (sodium/potassium/chloride transporter), member 2 |
| 7s1z.1.A | 12.55 | monomer | - | HHblits | EM | NA | 0.25 | 0.50 | Solute carrier family 12 member 2 |
| 7ccs.1.B | 11.76 | monomer | - | HHblits | EM | NA | 0.25 | 0.50 | Consensus mutated Anionic Amino Acid Transporter Light Chain, Xc- System |
| 6npl.1.B | 14.06 | monomer | - | HHblits | EM | NA | 0.26 | 0.49 | Solute carrier family 12 (sodium/potassium/chloride transporter), member 2 |
| 6pzt.1.A | 12.55 | monomer | - | HHblits | EM | NA | 0.25 | 0.50 | Solute carrier family 12 member 2 |
| 8a6l.1.B | 11.76 | monomer | - | HHblits | EM | NA | 0.25 | 0.50 | Large neutral amino acids transporter small subunit 2 |
| 7sfl.1.A | 12.40 | monomer | - | HHblits | EM | NA | 0.25 | 0.49 | Solute carrier family 12 member 2 |
| 6npl.1.A | 14.06 | monomer | - | HHblits | EM | NA | 0.26 | 0.49 | Solute carrier family 12 (sodium/potassium/chloride transporter), member 2 |
| 6nph.1.A | 12.25 | monomer | - | HHblits | EM | NA | 0.25 | 0.49 | Solute carrier family 12 (sodium/potassium/chloride transporter), member 2 |
| 3lrb.1.A | 16.06 | homo-dimer | 0.08 | HHblits | X-ray | 3.61Å | 0.26 | 0.49 | Arginine/agmatine antiporter |
| 8fhn.1.A | 12.65 | monomer | - | HHblits | EM | NA | 0.25 | 0.49 | Solute carrier family 12 member 2,Solute carrier family 12 member 3 chimera |
| 8fhp.1.A | 12.65 | monomer | - | HHblits | EM | NA | 0.25 | 0.49 | Solute carrier family 12 member 2,Solute carrier family 12 member 3 chimera |
| 7n3n.1.B | 12.60 | monomer | - | HHblits | EM | NA | 0.25 | 0.50 | Solute carrier family 12 member 2 |
| 8fho.1.B | 12.65 | monomer | - | HHblits | EM | NA | 0.25 | 0.49 | Solute carrier family 12 member 2,Solute carrier family 12 member 3 chimera |
| 7mxo.1.B | 13.36 | monomer | - | HHblits | EM | NA | 0.25 | 0.48 | Solute carrier family 12 member 2 |
| 7sfl.1.B | 12.40 | monomer | - | HHblits | EM | NA | 0.25 | 0.49 | Solute carrier family 12 member 2 |
| 7n3n.1.A | 12.60 | monomer | - | HHblits | EM | NA | 0.25 | 0.50 | Solute carrier family 12 member 2 |
| 8fhn.1.B | 12.65 | monomer | - | HHblits | EM | NA | 0.25 | 0.49 | Solute carrier family 12 member 2,Solute carrier family 12 member 3 chimera |
| 6jmq.1.A | 12.94 | monomer | - | HHblits | EM | NA | 0.26 | 0.50 | Large neutral amino acids transporter small subunit 1 |
| 7mxo.1.A | 13.36 | monomer | - | HHblits | EM | NA | 0.25 | 0.48 | Solute carrier family 12 member 2 |
| 7d10.1.A | 12.60 | monomer | - | HHblits | EM | NA | 0.25 | 0.50 | Solute carrier family 12 member 2 |
| 8fht.1.A | 12.90 | monomer | - | HHblits | EM | NA | 0.25 | 0.48 | Solute carrier family 12 member 3 |
| 7cmh.1.B | 11.86 | monomer | - | HHblits | EM | NA | 0.25 | 0.49 | Large neutral amino acids transporter small subunit 2 |
| 6yup.1.C | 9.45 | monomer | - | HHblits | EM | NA | 0.25 | 0.50 | b(0,+)-type amino acid transporter 1 |
| 7b00.1.A | 11.76 | monomer | - | HHblits | EM | NA | 0.26 | 0.50 | Large neutral amino acids transporter small subunit 2 |

  
The table above shows the top 50 filtered templates. A further 92 templates were found which were considered to be less suitable for modelling than the filtered list.  
2jln.1.A, 2x79.1.A, 2xq2.1.A, 2xq2.1.B, 3dh4.1.A, 4d1a.1.A, 4d1d.1.A, 4dji.1.A, 4dji.2.A, 4djk.1.A, 4djk.2.A, 4m48.1.A, 4us3.1.A, 4us4.1.A, 4xnu.1.A, 4xnx.1.A, 4xp1.1.A, 4xp4.1.A, 4xpa.1.A, 4xpf.1.A, 4xpg.1.A, 4xpt.1.A, 5kte.1.A, 5m8a.1.A, 5m8j.1.A, 5m94.1.A, 5m95.2.B, 5nva.1.A, 6c3i.1.A, 6cse.1.C, 6d91.1.A, 6d9w.1.A, 6dzv.1.A, 6dzz.1.A, 6irs.1.B, 6irt.1.B, 6kkr.1.A, 6m17.1.D, 6m18.1.C, 6m1d.1.A, 6m1y.1.A, 6m22.1.A, 6m23.1.A, 6s3k.1.A, 6ukn.1.A, 6vrh.1.A, 6vrk.1.A, 6vrl.1.A, 6wti.1.D, 6y5r.1.A, 6y5v.1.A, 6zbv.1.A, 7ain.1.A, 7aip.1.A, 7air.1.A, 7cub.1.D, 7d14.1.A, 7d8z.1.A, 7d90.1.A, 7d99.1.A, 7dsk.1.B, 7dsn.1.B, 7dsq.1.B, 7li7.1.A, 7mgw.1.A, 7ngb.1.A, 7phq.1.C, 7qia.1.A, 7qoa.1.A, 7sl9.1.A, 7sla.1.A, 7tth.1.A, 7tti.1.A, 7uuy.1.A, 7uuz.1.A, 7uv0.1.A, 7vsi.1.A, 7xmc.1.D, 7xmd.1.D, 7y75.1.A, 7y76.1.A, 7ynj.1.A, 7ynk.1.A, 8b70.1.B, 8b71.1.A, 8e5s.1.A, 8e6h.1.A, 8e6i.1.A, 8e6l.1.A, 8e6m.1.A, 8e6n.1.A, 8ont.1.A

Swiss Institute of Bioinformatics
Contact Us
